# Supplementary material for: Desmopressin for reversal of Antiplatelet drugs in Stroke due to Haemorrhage (DASH): protocol for a phase II double-blind randomised controlled feasibility trial
Source: BMJ Open. 2020 Nov 10;10(11):e037555. doi: 10.1136/bmjopen-2020-037555 (PMC7656949; doi:10.1136/bmjopen-2020-037555)
Supplement: Supplementary data [file bmjopen-2020-037555supp001.pdf]

***(Form to be printed on local headed paper)***

# PARTICIPANT CONSENT FORM

**(**Final version 2.0: 26 August 2019**)**

**Title of Study: Desmopressin for reversal of Antiplatelet drugs in Stroke due to Haemorrhage (DASH)**

**IRAS Project ID: 233744.** **CTA ref : 03057/0070/001-0001**

**Name of Researcher**:

**Name of Participant:**

**Please initial box**

1. I confirm that I have read and understand the information sheet version number 2.0: 26 August 2019 for the above study and have had the opportunity to ask questions. ☐
2. I understand that my participation is voluntary and that I am free to withdraw at any time, without giving any reason, and without my medical care or legal rights being affected. I understand that should I withdraw, then the information collected so far cannot be erased and that this information may still be used in the project analysis. ☐
3. I understand that relevant sections of my medical notes and data collected in the study may be looked at by authorised individuals from the University of Nottingham, the research group and regulatory authorities where it is relevant to my taking part in this study. I give permission for these individuals to have access to these records and to collect, store, analyse and publish information obtained from my participation in this study. I understand that my personal details will be kept confidential. ☐
4. I understand and agree that blood samples will be taken for analysis to monitor the effects of treatment given in DASH. Samples will be destroyed after analysis. ☐
5. I understand that the information held and maintained by the NHS Digital and other central UK NHS bodies may be used to help contact me or provide information about my health status. ☐
6. I agree to my GP being informed of my participation in this study and providing information about my health status and contact details if needed. ☐
7. I agree to take part in the above study. ☐

| Name of Participant | Date | Signature |
|---|---|---|

| Name of Person taking consent | Date | Signature |
|---|---|---|

**PARTICIPANT CONSENT FORM - (**Final version 2.0: 26 August 2019**)**

3 copies: 1 for participant, 1 for the project notes and 1 for the medical notes
