# Supplementary material for: Desmopressin for reversal of Antiplatelet drugs in Stroke due to Haemorrhage (DASH): protocol for a phase II double-blind randomised controlled feasibility trial
Source: BMJ Open. 2020 Nov 10;10(11):e037555. doi: 10.1136/bmjopen-2020-037555 (PMC7656949; doi:10.1136/bmjopen-2020-037555)
Supplement: Supplementary data [file bmjopen-2020-037555supp002.pdf]

Local Letterhead to be added

Participant Information Sheet
(Final version 2.0: 26 August 2019)

IRAS Project ID: 233744

Title of Study: Desmopressin for reversal of Antiplatelet drugs in Stroke due to Haemorrhage (DASH)

Name of Chief Investigator: Prof Nikola Sprigg
Local Researcher(s): xxxxxxxxxxxxxxxxxxxxxxxx

We would like to invite you to take part in our research study. Before you decide we would like you to understand why the research is being done and what it would involve for you. One of our team will go through the information sheet with you and answer any questions you have. Talk to others about the study if you wish. Ask us if there is anything that is not clear, or if you would like more information.

What is the purpose of the study?

When someone has a stroke caused by bleeding into the brain (haemorrhagic stroke) permanent brain damage can occur and result in long term disability. There is also a chance that the bleeding can increase, which may cause worse disability or be life threatening. This happens in approximately 20-30% of haemorrhagic stroke patients. Bleeding is worse for patients taking drugs such as aspirin or clopidogrel (anti-platelet drugs).

At present, there is no available treatment that is effective at reducing the bleeding in the brain and improving the recovery. New treatments are being developed to treat stroke, but it can be very hard to test whether they work in the first few hours because often patients take longer than this to get to hospital and have investigations such as brain scanning. Also, some treatments are not suitable for all patients. This can make testing new treatments difficult.

In this trial, we want to test whether it is possible to give a drug (desmopressin) to patients within 24 hours after a haemorrhagic stroke. Continued or increased bleeding into the brain (so called haematoma expansion) is not uncommon in the first hours and days following a haemorrhagic stroke and increases the risk of the patient not recovering fully and being left with some disability. Stopping the bleeding in the first hours and days after stroke with medications might help patients to recover better. The treatment we are testing is a drug that encourages blood to clot - to stop bleeding. We hope that we will be able to show that giving the drug may reduce the chances of dying and being left with disability after a haemorrhagic stroke. This drug is not given routinely after stroke.

We aim to assess in this trial what effect desmopressin has on bleeding after a haemorrhagic stroke. Desmopressin is a tried and tested drug in other medical conditions that acts quickly to help the blood to clot and stop bleeding.

In order to do a proper comparison, we need to give some people the active drug and some people a dummy (placebo) treatment. In this trial, the dummy treatment is salt water. Half of the patients in the trial will receive the drug desmopressin and half will have placebo treatment.

The data will help doctors decide whether blood thickening treatments like desmopressin can be used in patients with acute haemorrhagic strokes who are taking anti-platelet drugs to try to prevent death and improve recovery.

Why have I been invited?

You are being invited to take part because you have had a stroke caused by bleeding into the brain – this is called a haemorrhagic stroke. We are inviting 50 participants like you to take part in the study.

Do I have to take part?

It is up to you to decide whether or not to take part. If you do decide to take part you will be given this information sheet to keep and be asked to sign a consent form. If you decide to take part you are still free to withdraw at any time and without giving a reason. This would not affect your legal rights.

What will happen to me if I take part?

Your involvement in the study will last for 3 months. In this study, the treatment (either desmopressin or dummy) is given as a drip via a cannula (needle inserted into a vein, usually into the back of the hand), which will be given over approximately 20 minutes.

We select which treatment you receive randomly (like tossing a coin) because this is how most clinical trials are carried out. When we don't know whether a treatment is effective or not we need to test it against not getting that treatment (called a control). Using randomisation to put patients into treatment groups is the best way to get a true answer as to whether a treatment works or not. In this trial, it is important to test whether the drug desmopressin can be given at random in a trial.

The treatment (either desmopressin or dummy) will be given a drip as soon as possible once you have decided you wish to take part in the study. The treatment will be given via a drip over approximately 20 minutes. You will not know if you received the drug or the dummy. The treatment will be given once, and then the treatment will stop.

We will take 3 blood samples from the vein in your arm. One before we start the study treatment to look at your blood clotting status, one immediately after and another 24 hours after treatment to check the salt levels in your blood stream. Wherever possible these will be taken with any routine blood samples your doctor asks for.

During the next 7 days, a nurse will check your condition looking in particular for signs of side effects of the treatment. We will also repeat a brain scan the day after the treatment to assess effects of the treatment. The brain scan will last less than 5 minutes but you will need to go to the x-ray department for the brain scan, which may take approximately an hour.

We ask your permission to contact your GP or check with the NHS Information Centre to check on your condition three months after your stroke and to confirm your contact details. You will then be contacted for a telephone consultation with a member of the research team, this can also be conducted by postal questionnaire if you prefer. This is to check your condition at that time. It will involve asking how you are able to move around, about how you feel your life has been affected by the stroke and some brief memory tests. In order to make the final evaluation of the study as objective as possible, the person who telephones you will not know if you received the active treatment or not.

Other than described here, your treatment will be exactly the same as for all stroke patients.

Expenses and payments

Participants will not be paid to participate in the study. There will be no charge for the trial medication. Travel expenses will be offered for any visits incurred as a result of participation.

What are the possible disadvantages and risks of taking part?

Treatment with any drugs can result in possible side effects and the side effects from desmopressin are generally mild. They can include headache, abdominal pain, low blood pressure and dizziness. The drug can increase the risk of seizures but this is very rare.

However, because the treatment works by stopping bleeding there is a chance it can cause an increase in blood clot formation. This can occur in the legs (deep vein thrombosis, DVT) or the lungs (Pulmonary embolism, PE) and is potentially very serious and maybe even life threatening. If you have previously suffered from blood clots in the legs or lungs you may not be able to participate in this study.

In 65 previous studies where desmopressin has been used to reduce bleeding during operations, desmopressin was safe. There was no increase in serious side effects, such as blood clots, in the patients who were treated with desmopressin.

Because desmopressin is already routinely used in a number of bleeding conditions, we expect the potential benefit of the drug (stopping bleeding in to the brain) to outweigh the low risk of serious side effects (such as blood clots). However, we do not know this for certain and will monitor all participants closely for side effects.

You must inform your doctor or member of the research team if you feel you have had a reaction to the medication.

We will take 3 blood samples from the vein in your arm, this can cause mild discomfort/pain and slight bruising.

You will have an extra CT brain scan performed as part of this trial. This is exactly the same as the CT scan that you had when you first came to hospital. The scan itself takes less than 5 minutes and does not involve any injections. The scan uses x-rays, which in large amounts can be harmful, but for this extra CT head scan the additional risk to you from the scan has been judged to be extremely small and is comparable with the annual risk of dying from an accident in the home.

You will need to be followed up by the research team for 3 months after starting the study.

What are the possible benefits of taking part?

Your participation in this study may reduce the symptoms of your haemorrhagic stroke or improve long-term recovery. However, we cannot promise the study will help you, and participation is voluntary. The information we get from your involvement may benefit other people who may have a stroke in the future.

What happens when the research study stops?

We aim to treat 50 patients in this study from the UK. When it has finished, we will look at the data and decide whether the treatment could be used for more patients with haemorrhagic stroke. We may not directly tell you or your relative the results of the study, but they will be published in a journal where they can be read.

Desborough MJR, *et al. BMJ Open* 2020; 10:e037555. doi: 10.1136/bmjopen-2020-037555

Will my taking part in the study be kept confidential?

We will follow ethical and legal practice and all information about you will be handled in confidence.

If you join the study, we will use information collected from you [and your medical records] during the course of the research. This information will be kept **strictly confidential**, stored in a secure and locked office, and on a password protected database at the University of Nottingham. Under UK Data Protection laws the University is the Data Controller (legally responsible for the data security) and the Chief Investigator of this study (named above) is the Data Custodian (manages access to the data). This means we are responsible for looking after your information and using it properly. Your rights to access, change or move your information are limited as we need to manage your information in specific ways to comply with certain laws and for the research to be reliable and accurate. To safeguard your rights we will use the minimum personally – identifiable information possible.

You can find out more about how we use your information and to read our privacy notice at: https://www.nottingham.ac.uk/utilities/privacy.aspx. A hard copy can be made available on request.

The data collected for the study will be looked at and stored by authorised persons from the University of Nottingham who are organising the research. They may also be looked at by authorised people from regulatory organisations to check that the study is being carried out correctly. All will have a duty of confidentiality to you as a research participant and we will do our best to meet this duty.

Where possible information about you which leaves the site will have your name and address removed and a unique code will be used so that you cannot be recognised from it. However sometimes we need to ensure that we can recognise you to link the research data with your medical records so in these instances we will need to know your name and date of birth. We will also need this information if we need to follow up your medical records as part of the research, where we may need to ask the Government services that hold medical information about you (such as NHS Digital, the Office for National Statistics, among others) to provide this information to us. By signing the consent form you agree to the above.

Your contact information will be kept by the University of Nottingham for 6 months after the end of the study so that we are able to contact you about the findings of the study and possible follow-up studies (unless you advise us that you do not wish to be contacted). This information will be kept separately from the research data collected and only those who need to will have access to it. All other data (research data) will be kept securely for 7 years. After this time your data will be disposed of securely. During this time all precautions will be taken by all those involved to maintain your confidentiality, only members of the research team given permission by the data custodian will have access to your personal data.

In accordance with the University of Nottingham's, the Government's and our funders' policies we may share our research data with researchers in other Universities and organisations, including those in other countries, for research in health and social care. Sharing research data is important to allow peer scrutiny, re-use (and therefore avoiding duplication of research) and to understand the bigger picture in particular areas of research. Data sharing in this way is usually anonymised (so that you could not be identified) but if we need to share identifiable information we will seek your consent for this and ensure it is secure. You will be made aware then if the data is to be shared with

countries whose data protection laws differ to those of the UK and how we will protect your confidentiality.

Although what you say to us is confidential, should you disclose anything to us which we feel puts you or anyone else at any risk, we may feel it necessary to report this to the appropriate persons.

What will happen if I don't want to carry on with the study?

Your participation is voluntary and you are free to withdraw at any time, without giving any reason, and without your legal rights being affected. If you withdraw we will no longer collect any information about you or from you. We will keep the information about you that we have already obtained as we are not allowed to tamper with study records and this information may have already been used in some analyses and may still be used in the final study analyses.

Involvement of the General Practitioner/Family doctor (GP)

If you are enrolled in the study we will inform your General Practitioner.

What will happen to any samples I give?

The samples will be stored with a code unique to you and securely at the University of Nottingham and at The Oxford Haemophilia and Thrombosis Centre under the University's Human Tissue Research Licence (no 12265). At the end of the study samples will be destroyed in accordance with the Human Tissue Act guidelines.

Will any genetic tests be done?

No, we will not be collecting any genetic samples.

What will happen to the results of the research study?

The results of the research may be published. If so, this will be in a medical journal. You will not be identified in any report.

Who is organising and funding the research?

This research is being organised by the University of Nottingham and is being funded by NIHR RfPB.

Who has reviewed the study?

All research in healthcare is looked at by independent group of people, called a Research Ethics Committee, to protect your interests. This study has been reviewed and given favourable opinion by the local Ethics Committee [*NHS HRA East Midlands – Nottingham 2*]

Who should be contacted if there is a problem?

If you have a concern about any aspect of this study, you should ask to speak to the researchers who will do their best to answer your questions. The researchers' contact details are given at the end of this information sheet. If you remain unhappy and wish to complain formally, you can do this by contacting the [please provide below the contact details of PALS for the local hospital]

In the event that something does go wrong and you are harmed during the research and this is due to someone's negligence then you may have grounds for a legal action for compensation against the University of Nottingham but you may have to pay your legal costs. The normal National Health Service complaints mechanisms will still be available to you.

Further information and contact details

Trial / Study Coordinating Centre:
Division of Clinical Neuroscience, Stroke
University of Nottingham
Hucknall Road
Nottingham
NG5 1PB
Phone: 0115 8231770
Chief Investigator:
Prof Nikola Sprigg
Phone:0115 8231765

Contact details of PALS for the local hospital:
