# Supplementary material for: Desmopressin for reversal of Antiplatelet drugs in Stroke due to Haemorrhage (DASH): protocol for a phase II double-blind randomised controlled feasibility trial
Source: BMJ Open. 2020 Nov 10;10(11):e037555. doi: 10.1136/bmjopen-2020-037555 (PMC7656949; doi:10.1136/bmjopen-2020-037555)
Supplement: Supplementary data [file bmjopen-2020-037555supp003.pdf]

University of Nottingham
UK | CHINA | MALAYSIA

DASH: Desmopressin for reversal of Antiplatelet drugs in Stroke due to Haemorrhage

IRAS Project ID: 233744 **CTA ref: 03057/0070/001-0001**

Name of Researcher: ______________________________

Name of Participant: ______________________________

I confirm that I have been given a copy of the Patient Information Sheet (Version 2.0 dated 11 Jan 2019) and I agree that I /my relative or friend / this stroke patient (delete as appropriate)

- Will take part in the DASH study
- For my medical records to be accessed, blood samples to be taken and stored and brain scans used by the study team
- To be followed up at 3 months
- For my GP to be informed
- For my contact details to be collected and used for the purpose of the study
- For my confidential data to be used in further research analysis about ICH.

I understand that I am free to withdraw from the study at any point without giving a reason.

Patient consent – to be completed if participant has capacity to consent

Name of Participant	Date	Signature
Name of Person taking consent	Date	Signature
Name of Witness if participant unable to physically sign	Date	Signature

Personal or professional nominee consent - to be completed if participant does not have capacity to consent

Name of Person giving nominee consent	Date	Signature
Relationship to patient (please tick):	Relative/carer/friend ☐	Healthcare Professional ☐
Name of Person taking consent	Date	Signature
**Telemedicine used** (please tick if Yes) ☐		
Name of Witness if consent taken [illegible]	Date	Signature

Short patient information sheet – pictorial - Final v2.0 – 11 Jan 2019

University of Nottingham
UK | CHINA | MALAYSIA

DASH

DASH Research Study – Information Sheet

Final version 2.0 – 11 Jan 2019

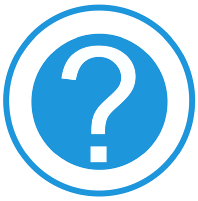	**What is this about?**<br>• We want to know if you would like you to take part in a research study called DASH. DASH will test the effect of the drug called desmopressin that may stop bleeding by reversing the effect of blood thinning medication.<br>• Research staff will discuss the study with you and can answer any questions you may have. If you are not well enough we will try to ask your family or friend. If they are not present an independent doctor will decide for you on your behalf.<br>• Taking part in the study is voluntary; you don't have to take part.
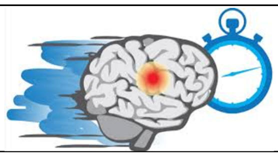	**Why are we asking you to take part in the study?**<br>• You have had a stroke caused by bleeding in the brain and you have been taking blood thinning medication.
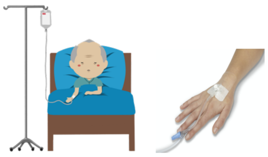<br>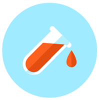<br>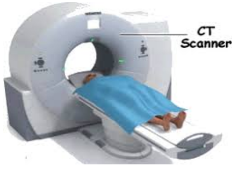 	**If you take part:**<br>• You will receive all the care and treatments you would normally receive.<br>• The medical team will give you a drip into your vein via a small needle, which takes 20 minutes.<br>• Half the people in this study will get a drip containing desmopressin and half will get a drip containing no drug (placebo).<br>• Which drip you are given is decided by chance (like flipping a coin). You won't know which drip you will have been given.<br>• You will have three blood tests: one before the drip, one immediately after and another one on the next day to monitor results of the drip.<br>• You will have an extra head scan at some point in the next 2 days to monitor the bleeding. The study team will have copies of your brain scans.
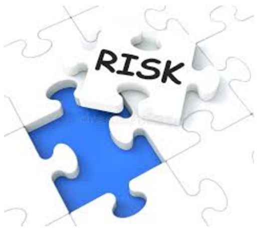 	**Risks**<br>The drug, desmopressin, has been given safely to thousands of patients with inherited bleeding problems.<br>• The drug can cause mild side effects: headache, nausea and vomiting, which can all be easily treated. Very rarely it can cause more serious sides effects and we will monitor closely for this.<br>• The extra head scan has around the same amount of radiation as living for a year in the UK.
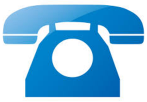	**90 days after your stroke:**<br>• A researcher will call you to see how you are, if you have had any problems and how well you have recovered.<br>• If you are not well enough to talk we will try to ask your family, friend or GP.
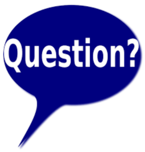 	**During the study:**<br>• If you have any questions then please ask.<br>• You may decide you do not want to take part at any time. This will not affect your care now or in the future.<br>• All the information we hold about you (including brain scans) will be kept in the strictest confidence.

Short patient information sheet – pictorial - Final v2.0 – 11 Jan 2019
