# Supplementary material for: Desmopressin for reversal of Antiplatelet drugs in Stroke due to Haemorrhage (DASH): protocol for a phase II double-blind randomised controlled feasibility trial
Source: BMJ Open. 2020 Nov 10;10(11):e037555. doi: 10.1136/bmjopen-2020-037555 (PMC7656949; doi:10.1136/bmjopen-2020-037555)
Supplement: Author's manuscript [file bmjopen-2020-037555.draft_revisions.pdf]

BMJ Open is committed to open peer review. As part of this commitment we make the peer review history of every article we publish publicly available.

When an article is published we post the peer reviewers' comments and the authors' responses online. We also post the versions of the paper that were used during peer review. These are the versions that the peer review comments apply to.

The versions of the paper that follow are the versions that were submitted during the peer review process. They are not the versions of record or the final published versions. They should not be cited or distributed as the published version of this manuscript.

BMJ Open is an open access journal and the full, final, typeset and author-corrected version of record of the manuscript is available on our site with no access controls, subscription charges or pay-per-view fees (http://bmjopen.bmj.com).

If you have any questions on BMJ Open's open peer review process please email info.bmjopen@bmj.com

BMJ Open

Desmopressin for reversal of Antiplatelet drugs in Stroke due to Haemorrhage (DASH): protocol for a phase II double blind randomised controlled trial

Journal:	*BMJ Open*
Manuscript ID	bmjopen-2020-037555
Article Type:	Protocol
Date Submitted by the Author:	07-Feb-2020
Complete List of Authors:	Desborough, Michael; Guy's and Saint Thomas' Hospitals NHS Trust, Haemophilia and Thrombosis Centre; University of Oxford, NIHR Oxford Biomedical Research Centre, Radcliffe Department of Medicine<br>Salman, Rustam; University of Edinburgh Western General Hospital, Centre for Clinical Brain Sciences<br>Stanworth, Simon; Oxford University Hospitals NHS Foundation Trust, Department of Haematology; University of Oxford, NIHR Oxford Biomedical Research Centre, Radcliffe Department of Medicine<br>Havard, Diane; University of Nottingham, Division of Clinical Neuroscience<br>Brennan, Paul; University of Edinburgh Western General Hospital, Centre for Clinical Brain Sciences<br>Dineen, Rob; University of Nottingham, Division of Clinical Neuroscience; University of Nottingham, NIHR Nottingham Biomedical Research Centre<br>Coats, Timothy; University of Leicester, Department of Cardiovascular Sciences<br>Hepburn, Trish; University of Nottingham, Clinical Trials Unit<br>Bath, Philip; University of Nottingham, Division of Clinical Neuroscience<br>Sprigg, Nikola; University of Nottingham, Division of Clinical Neuroscience
Keywords:	Stroke medicine < INTERNAL MEDICINE, Bleeding disorders & coagulopathies < HAEMATOLOGY, Anticoagulation < HAEMATOLOGY, Stroke < NEUROLOGY, Clinical trials < THERAPEUTICS

SCHOLARONE™
Manuscripts

I, the Submitting Author has the right to grant and does grant on behalf of all authors of the Work (as defined in the below author licence), an exclusive licence and/or a non-exclusive licence for contributions from authors who are: i) UK Crown employees; ii) where BMJ has agreed a CC-BY licence shall apply, and/or iii) in accordance with the terms applicable for US Federal Government officers or employees acting as part of their official duties; on a worldwide, perpetual, irrevocable, royalty-free basis to BMJ Publishing Group Ltd ("BMJ") its licensees and where the relevant Journal is co-owned by BMJ to the co-owners of the Journal, to publish the Work in this journal and any other BMJ products and to exploit all rights, as set out in our licence.

The Submitting Author accepts and understands that any supply made under these terms is made by BMJ to the Submitting Author unless you are acting as an employee on behalf of your employer or a postgraduate student of an affiliated institution which is paying any applicable article publishing charge ("APC") for Open Access articles. Where the Submitting Author wishes to make the Work available on an Open Access basis (and intends to pay the relevant APC), the terms of reuse of such Open Access shall be governed by a Creative Commons licence – details of these licences and which Creative Commons licence will apply to this Work are set out in our licence referred to above.

Other than as permitted in any relevant BMJ Author's Self Archiving Policies, I confirm this Work has not been accepted for publication elsewhere, is not being considered for publication elsewhere and does not duplicate material already published. I confirm all authors consent to publication of this Work and authorise the granting of this licence.

For peer review only

Long title: Desmopressin for reversal of Antiplatelet drugs in Stroke due to Haemorrhage (DASH): protocol for a phase II double blind randomised controlled trial

Short title: DASH trial

Authors: Michael J R Desborough[1], Rustam Al-Shahi Salman[2], Simon J Stanworth[3,4,5], Diane Havard[6], Paul M Brennan[2], Robert A Dineen[6], Tim J Coats[7], Trish Hepburn[8], Philip M Bath[6], Nikola Sprigg[6]; DASH trial investigators

[1]Haemophilia and Thrombosis Centre, St Thomas' Hospital, London, [2]Centre for Clinical Brain Sciences, University of Edinburgh, Edinburgh, [3]Transfusion Medicine, NHS Blood and Transplant, Oxford, [4]Department of Haematology, Oxford University Hospitals NHS Foundation Trust, [5]Radcliffe Department of Medicine, University of Oxford, and NIHR Oxford Biomedical Research Centre, [6]Division of Clinical Neuroscience, University of Nottingham, Nottingham and Nottingham Biomedical Research Centre [7]Department of Cardiovascular Sciences, University of Leicester, Leicester, [8]Clinical Trials Unit, University of Nottingham, Nottingham, United Kingdom

Corresponding Author:

Professor Nikola Sprigg

Professor of Stroke Medicine

Room B60 Clinical Sciences Building

Nottingham City Hospital

Hucknall Road

Nottingham

NG5 1PB

United Kingdom

Phone +44 (0) 115 823 1778

Fax +44 (0) 115 823 1767

Email: nikola.sprigg@nottingham.ac.uk

For peer review only

Abstract

Introduction

Intracerebral haemorrhage can be devastating and is a common cause of death and disability worldwide. Pre-stroke antiplatelet drug use is associated with a 27% relative increase in one-month case fatality compared to patients not using antithrombotic drugs. We aim to assess the feasibility of conducting a randomised controlled testing the safety and efficacy of desmopressin for patients with antiplatelet-associated intracerebral haemorrhage.

Methods and Analysis

We aim to include 50 patients within 24 hours of spontaneous intracerebral haemorrhage onset, associated with oral antiplatelet drug(s) use in at least the preceding seven days. Patients will be randomised (1:1) to receive intravenous desmopressin 20μg in 50 mls sodium chloride 0.9% infused over 20 minutes or matching placebo. We will mask participants, relatives and outcome assessors to treatment allocation. Feasibility outcomes include proportion of patients approached being randomised, number of patients receiving allocated treatment, rate of recruitment, and adherence to treatment and follow up. Secondary outcomes include change in intracerebral haemorrhage volume at 24 hours; hyponatraemia at 24 hours, length of hospital stay, discharge destination, early death less than 28 days, death or dependency at day 90, death up to day 90, serious adverse events (including thromboembolic events) up to day 90; disability (Barthel index, day 90), quality of life (EuroQol 5D (EQ-5D], day 90), cognition (telephone mini-mental state examination day 90), and health economic assessment (EQ-5D).

Ethics and dissemination

The DASH trial (ISRCTN6703837, NCT03696121; EudraCT 2018-001904-12) received ethical approval from the East Midlands - Nottingham 2 research ethics committee (18/EM/0184). The DASH trial is funded by NIHR RfPB grant: PB-PG-0816-20011. Trial results will be published in a peer reviewed academic journal and disseminated through academic conferences and through patient stroke support groups. Reporting will be in compliance with CONSORT recommendations.

For peer review only - http://bmjopen.bmj.com/site/about/guidelines.xhtml

Strengths and limitations of the study

- This is the first randomised controlled trial of desmopressin versus placebo for patients taking antiplatelet drugs with spontaneous intracerebral haemorrhage
- Robust methodology developed from large international multicentre trials in spontaneous intracerebral haemorrhage such as TICH-2 and PATCH
- Desmopressin and placebo are not identical so the person administering the intervention will not be masked to treatment allocation.
- All outcomes assessors are blinded to treatment allocation to minimize the risk of bias

For peer review only

Introduction

Haemorrhagic stroke

Spontaneous intracerebral haemorrhage (SICH) caused approximately 3 million deaths worldwide in 2015[1]. Two-thirds of survivors are left dependent on others[2]. Roughly one third of patients are taking antiplatelet drugs at the time of SICH in high-income countries, and this proportion has been increasing over time[3]. Pre-stroke antiplatelet drug use is associated with a 27% relative increase in one-month case fatality compared to patients not using antithrombotic drugs[4]. Despite development of effective treatments for ischaemic stroke (thrombolysis, thrombectomy, aspirin, hemicraniectomy) there is no proven effective drug treatment for SICH, although blood pressure lowering might improve outcome[5].

Haematoma expansion

Outcome after SICH is closely related to haematoma growth (expansion) which is associated with worse outcome (death and disability)[6]. Use of antiplatelet drugs or anticoagulants, time from onset of symptoms to baseline imaging, and intracerebral haemorrhage volume on baseline imaging are independent predictors of haematoma expansion[7].

Platelet transfusion, tranexamic acid and recombinant activated factor VII have not been shown to improve outcomes after anti-platelet associated intracerebral haemorrhage, raising the need to consider alternatives [8].

Desmopressin

Desmopressin is a licensed pro-haemostatic drug that can be administered intravenously, subcutaneously, intranasally or orally and is used in a number of inherited bleeding conditions to treat or prevent bleeding[9-11]. Platelet function remains inhibited for 5-7 days after stopping antiplatelet drugs. Desmopressin stimulates release of Von Willebrand Factor (VWF) and factor VIII from endothelial Weibel-Palade bodies. VWF is responsible for platelet adhesion to collagen and may also bind platelets through their glycoprotein IIb/IIIa receptors [12], so increased levels of VWF have the potential to compensate for the platelet function defect

For peer review only

associated with antiplatelet drugs. Desmopressin may also increase the formation of procoagulant platelets [13].

Desmopressin is recommended for the reversal of antiplatelet drugs in guidelines from the USA[14] but not in UK guidelines[15]. In a recent meta-analysis evaluating desmopressin for reversal of antiplatelet drugs for patients undergoing cardiac surgery, desmopressin was found to reduce blood loss, transfusion requirements and the need for a further operation due to bleeding[16].

There are potential risks with use of desmopressin, including tachycardia hypotension, hyponatraemia and hyponatraemic seizures. A meta-analysis of 65 surgical randomised controlled trials, comparing desmopressin to placebo found no significant difference in rates of ischaemic stroke, myocardial infarction or venous thromboembolism[17].

Desmopressin has been tested in SICH in four case series where it appears to be safe[18-21]. However the series were small, non-randomised and did not have a placebo control arm, so it is not possible to make a clear assessment of the benefits and harms of administering desmopressin for these patients. American neurocritical care guidelines for the treatment of intracranial haemorrhage recommend consideration of 0.4 mcg/kg desmopressin for treatment of antiplatelet-associated sICH [14], although the level of evidence supporting this recommendation was recognised as very uncertain .

Searches of clinicaltrials.gov and World Health Organization International Trials Clinical Registry Platform found no other ongoing randomised controlled trials of desmopressin versus placebo for reversal of anti-platelet drugs in stroke due to haemorrhage.

For peer review only

Primary Objective

To assess the feasibility of randomising, administering the intervention, and completing follow-up for patients treated with desmopressin or placebo to inform a definitive trial.

Methods and analysis

DASH will be a multicentre double-blind randomised placebo-controlled, parallel group phase II feasibility trial. Participants will be enrolled by investigators from emergency departments or acute stroke units from 10 hospital sites in the United Kingdom. Patient flow though the trial is summarised in Figure 1.

Inclusion criteria:

- Adults (≥18 years)
- Confirmed SICH on imaging
- Randomised less than 24 hours from onset of symptoms [or from when last seen free of stroke symptoms]
- Prescribed and thought to be taking a daily oral antiplatelet drug in the preceding seven days (cyclooxygenase inhibitors, phosphodiesterase inhibitors or $P2Y_{12}$ inhibitors).

Exclusion criteria:

- Known secondary causes of SICH (aneurysmal subarachnoid haemorrhage or haemorrhage due to transformation of infarction, thrombolytic drug, venous thrombosis, arteriovenous malformation or tumour)
- Patients at risk of fluid retention
- Systolic blood pressure less than 90mmHg
- Known drug eluting stent in previous 3 months
- Allergy to desmopressin
- Pregnant or breast-feeding
- Life-expectancy less than 4 hours or planned for palliative care only
- Glasgow coma scale less than 5
- Modified Rankin scale (mRS) more than 4.

For peer review only - http://bmjopen.bmj.com/site/about/guidelines.xhtml

Consent:

Patients with capacity to give consent will be approached directly. If this is not possible, a relative or close friend will be approached for consent, if this is not possible then a professional not associated with the trial will be approached for proxy consent. Patients will be approached again for consent when they regain capacity.

Randomisation:

Participants will be randomised centrally using a secure internet site in real-time. Treatment allocation will be concealed from all staff and patients involved in the trial. Randomisation involves minimisation on key prognostic risk factors: age (70 years or more); sex (male); time since onset (3 hours or more); systolic blood pressure (170mmHg or more); and presence (or no information on presence or absence) of intraventricular haemorrhage. This approach ensures concealment of allocation, minimises differences in key baseline prognostic variables, and slightly improves statistical power [22]. Randomisation will allocate a number corresponding to a treatment pack and the participant will receive treatment from the allocated numbered pack. It was considered not possible to mask/blind personnel preparing the injection for administration to the treatment allocation because the ampoules of active and placebo injections are different and carry the manufacturer's identifying information. Clinicians, patients and outcome assessors (clinical, radiological and haematological assessors) will be blinded to treatment allocation.

Intervention:

Patients will be randomised to either 20 µg intravenous desmopressin (DDAVP, 4µg/ml 1ml glass ampoules, Ferring Pharmaceuticals) or placebo of intravenous Sodium Chloride 0.9%. Either desmopressin 20µg (5x 4µg/ml) will be added to 50ml Sodium Chloride 0.9% and infused over 20 minutes, or three 2ml Sodium Chloride 0.9% will be added to 50ml Sodium Chloride 0.9% and infused over 20 minutes. The investigational medicinal product (IMP) will be stored between 2◦C and 8◦C until use. All patients will receive all usual standard care including venous thromboembolism prophylaxis with intermittent pneumatic compression devices, blood pressure lowering therapy and neurosurgery if appropriate.

For peer review only - http://bmjopen.bmj.com/site/about/guidelines.xhtml

We will administer a single dose as the risk of continued bleeding and haematoma expansion after ICH is greatest in the first few hours. High levels of Factor VIII and VWF are maintained for 6–8 h after intravenous infusion therefore we believe a repeated dose at 12-24 hours would not be indicated. This dose regime (0.4 micrograms per kilogram body weight administered by intravenous infusion) is recommended in American neurocritical care guidelines for the treatment of intracranial haemorrhage[14], although the level of evidence supporting this recommendation was recognised as very uncertain .

Assessments:

Participants' age, sex, medical history, antiplatelet drug use, intracerebral haemorrhage location, intraventricular haemorrhage and mRS will be assessed at baseline by local investigators. Participants will be reviewed again on day 2, hospital discharge and on day 90 to gather information on interventions, adverse events, discharge date and discharge destination.

At day 90 central assessors trained in the appropriate questionnaires and masked to treatment allocation will follow up each patient by telephone. Barthel index, mRS, EuroQOL, cognition (mini-mental state examination) and serious adverse events will be assessed by telephone.

Brain imaging (CT head) will be undertaken as part of routine care before enrolment. A second research CT head scan will be performed 24 hours after treatment with the IMP to measure parenchymal haematoma volume. Haematoma volume will be measured centrally by trained assessors masked to treatment allocation using semi-automated segmentation. We will assess change in haematoma volume at 24 hours, and proportion of patients with haematoma expansion, defined as an absolute increase of greater than 6ml or relative growth of greater than 33%.

To assess baseline platelet function, platelets will be stimulated with arachidonic acid (which is inhibited by aspirin) or adenosine diphosphate (which is inhibited by P2Y12 inhibitors). Platelet cell surface P-selectin expression (a measure of platelet

For peer review only

activation) will then be measured using a standardised assay [Heptinstall; patent pending (PTC/GB2008/050169)] to assess retrospectively whether patients were taking antiplatelet drugs, as previously described [23]. Von Willebrand factor (VWF) antigen, VWF activity and factor VIII (1-stage) will be measured centrally by assessors masked to treatment allocation. Blood samples will be drawn into 0.109M citrate and centrifuged at 2500g for 15 minutes. Platelet poor plasma will be aspirated and stored at -80◦C at each site and then transferred to the central laboratory at the end of the trial for analysis. These tests will be performed for research only and the results will not be fed back to the clinical team. Serum sodium will be assessed locally 24 hours after administration of the IMP and these results will be available to the clinical team.

Outcomes:

This is a feasibility trial and the primary outcomes are based around feasibility: Number of eligible patients who receive allocated treatment; rate of eligible patients randomised; proportion of eligible patients approached; proportion of eligible patients randomised and reasons for non-randomisation; adherence to intervention; proportion of participants followed up to 90 days and reasons for loss to follow up; proportion of randomised participants with full outcome data available, and reasons for non-availability.

Secondary outcomes include change in intracerebral haemorrhage volume at 24 hours; hyponatraemia at 24 hours, length of hospital stay, discharge destination, early mortality less than 28 days, death or dependency at day 90, mortality up to day 90, serious adverse events (including thromboembolic events) up to day 90; disability (Barthel index, day 90), quality of life (EuroQol, day 90), cognition (telephone MMSE day 90), and health economic assessment (EQ-5D). Baseline platelet dysfunction will be measured and correlated with response to desmopressin; and change in factor VIII, VWF antigen and VWF activity will be assessed one hour after administration of desmopressin.

Adverse events:

All adverse events on Day 1 (including during infusion) and for the 24-hour period post dose will be collected. All adverse events will be assessed for seriousness,

For peer review only - http://bmjopen.bmj.com/site/about/guidelines.xhtml

expectedness and causality by adjudicators masked to treatment allocation. Serious adverse events will be categorised in accordance with the medical dictionary for regulatory authorities (MeDRA). As the IMP is administered once and has a short half-life, serious adverse events occurring within the first 7 days will be assessed for seriousness, expectedness and causality. In addition fatal SAEs and safety outcome events (fluid overload, hyponatraemia) will be reported until day 90.

Statistical analysis

Data will be analysed by a qualified statistician who is blinded to treatment allocation, using a validated software package. A statistical analysis plan (SAP) will be agreed prior to database lock and release of randomisation codes. The trial will be reported in accordance with CONSORT guidelines including the extension to pilot and feasibility trials, as appropriate [24-25]. This is a feasibility trial and the main analysis will be with descriptive statistics only. Counts will be summarised using N and %, and continuous variables will be summarised using means and standard deviations or medians and interquartile ranges depending on their distribution. Whilst some variables will be summarised by treatment group, no formal statistical comparisons will be made and any analyses will be considered purely exploratory. We will assess the feasibility of recruiting, treating and following up patients from 10 centres over one year. We will estimate a recruitment rate, treatment rate and follow-up rate. It is likely that a large definitive trial would be feasible if at least 50 participants were recruited into this study, that compliance with randomised treatment was high and that a high proportion of follow up data was available. Lower recruitment would not preclude progression if there was some evidence that the barriers to recruitment identified could be overcome. A decision about feasibility for a larger trial will be made in conjunction with the trial steering committee taking into account recruitment rates, differences between centres and other relevant information.

This is a feasibility trial and as such will have no formal assessment of efficacy. The proposed primary efficacy outcome in a definitive trial would be death or dependency at day 90, measured using the mRS. Shifts in the mRS will be summarised for this trial but no formal confirmatory statistical analyses will be performed. Similarly, all other efficacy variables will be summarised using descriptive statistics. Serious adverse

For peer review only

events will be summarised using descriptive statistics according to the treatment the participant received. Missing data will be reported. The investigation of this data and methods implemented to address the missing data, if appropriate, will be detailed in the SAP.

All available data will be used including overall numbers of patients presenting in clinic and screening data (where available). Where summaries by treatment group are provided, these will be based on an intention to treat population i.e. according to the treatment the participant was randomised to, with the exception of safety data. A safety population will be defined to summarise the safety data in this study where participants will be summarised according to the treatment they received irrespective of randomisation. Summaries of the number and proportion of participants who would form a per protocol population in a larger trial, and reasons for exclusion from a per protocol population will be provided to allow future planning. No other data will be summarised for this population.

Sample size and justification

Since this is a feasibility study with one of the objectives being to determine potential recruitment rates, a formal sample size calculation is not appropriate. If more than 50 participants were randomised from 10 centres in a 12 month period, it is likely that, assuming similar recruitment rates in additional centres, a larger study recruiting approximately 1200 participants in around 50 centres recruiting for 60 months would be feasible. Depending on the final sample size calculation for the definitive study, the number of centres and recruitment period could be determined using the information from the rates and patterns observed in the feasibility study. Information about set up times for centres will also better inform recruitment projections for a larger study. TICH-2 and other studies indicate approximately 25% of all people presenting with SICH are taking antiplatelet drugs [26].

Patient and public involvement

This study was developed in collaboration with, and is supported by Nottingham Stroke Research Partnership Group, made up of stroke survivors and carers. Members of the group have reviewed the proposed study, and in an iterative process,

For peer review only - http://bmjopen.bmj.com/site/about/guidelines.xhtml

commented on its design and conduct. In particular, the group has influenced the approach to taking informed consent within the study. In addition, the proposal was also reviewed by the Research to Understand Stroke due to Haemorrhage (RUSH) patient reference group in Edinburgh (http://www.ed.ac.uk/clinical-brain-sciences/research/diagnoses-diseasetargets/rush/rush-patient-reference-group).

Ethics and dissemination

The trial will be overseen by a trial steering committee. A trial management committee based at the Stroke Trials Unit in Nottingham, UK will be responsible for day-to-day conduct of the trial. An independent data monitoring committee will review the data at six months. Study data will be collected, monitored and analysed in Nottingham. The trial will be run in accordance with the principles of good clinical practice and the Declaration of Helsinki. The DASH trial has been granted ethics approval by the East Midlands - Nottingham 2 research ethics committee (18/EM/0184). The trial has been adopted in the UK by the National Institute for Health and Care Research (NIHR) clinical trial network and is registered as NCT03696121, ISRCTN67038373 and EudraCT 2018-001904-12.

Trial results will be published in a peer reviewed academic journal. Reporting will be in compliance with CONSORT[24-25] recommendations. When the study is complete summary findings will be posted on the patient support group website. Findings will also be presented at conferences such as the UK Stroke Forum, European Stroke Conference, World Stroke Congress, British Society for Haematology annual meeting, and European Congress on Thrombosis and Haemostasis.

Protocol amendments

In the initial version of the protocol, which was in place from the start of the trial until 30/11/2019, participants could be recruited if they could be randomised less than 12 hours from onset of symptoms [or from when last seen free of stroke symptoms]. In the updated version of the protocol, which came into effect on 01/12/2019, participants could be recruited if they could be randomised less than 24 hours from onset of symptoms [or from when last seen free of stroke symptoms]

For peer review only - http://bmjopen.bmj.com/site/about/guidelines.xhtml

Discussion

This will be the first randomised controlled trial comparing desmopressin to placebo for patients with SICH who are taking antiplatelet drugs. SICH is a common cause of death and disability worldwide. Patients taking antiplatelet drugs are more likely to have haematoma expansion and to have poorer outcomes than those who are not taking an antiplatelet drug. Early treatment with drugs to reduce the effect of antiplatelet drugs may reduce haematoma expansion. Desmopressin is a promising drug, which is commonly used in the treatment of inherited bleeding disorders and may reduce the effects of antiplatelet drugs.

In this trial we aim to determine if it is feasible to administer desmopressin within 24 hours to patients with SICH taking an antiplatelet drug. One potential barrier is that some patients will have delayed presentations to hospital. For those that reach hospital within 24 hours, we have aimed to make recruitment to the trial streamlined by minimising barriers to recruitment. This includes use of broad inclusion criteria and using a simple trial design. The design of this trial is based on the successful TICH-2[26] and PATCH[27] multicentre trials. The use of consent from a relative, close friend or professional representative is essential in this trial because the majority of patients will lack capacity.

If it proves feasible to run a trial of desmopressin compared to placebo for these patients then we aim to proceed to a large efficacy study. This will have an important impact on clinical practice. Desmopressin is widely available and inexpensive. It has the potential to reduce the risk of death and disability for patients with SICH who are taking antiplatelet drugs. Desmopressin could be rapidly adopted into clinical guidelines if it proves to reduce the risk of death or disability for patients with SICH.

For peer review only - http://bmjopen.bmj.com/site/about/guidelines.xhtml

References

1. GBD 2015 Mortality and Causes of Death Collaborators. Global, regional, and national life expectancy, all-cause mortality, and cause-specific mortality for 249 causes of death, 1980-2015: a systematic analysis for the Global Burden of Disease Study 2015. *Lancet* 2016;388:1459-544.
2. Royal College of Physicians. Sentinal Stroke National Audit Programme (SSNAP). 2016.
3. Lovelock CE, Molyneux AJ, Rothwell PM. Change in incidence and aetiology of intracerebral haemorrhage in Oxfordshire, UK, between 1981 and 2006: a population-based study. *Lancet Neurol* 2007;6:487-93.
4. Thompson BB, Bejot Y, Caso V, et al. Prior antiplatelet therapy and outcome following intracerebral hemorrhage: a systematic review. *Neurology* 2010;75:1333-42.
5. Anderson CS, Heeley E, Huang Y, et al. Rapid blood-pressure lowering in patients with acute intracerebral hemorrhage. *N Engl J Med* 2013;368:2355-65.
6. Steiner T, Bosel J. Options to restrict hematoma expansion after spontaneous intracerebral hemorrhage. *Stroke* 2010;41:402-9.
7. Al-Shahi Salman R, Frantzias J, Lee RJ, et al. Absolute risk and predictors of the growth of acute spontaneous intracerebral haemorrhage: a systematic review and meta-analysis of individual patient data. *Lancet Neurol* 2018;17:885-94
8. Al-Shahi Salman R, Law ZK, Bath PM, et al. Haemostatic therapies for acute spontaneous intracerebral haemorrhage. *Cochrane Database Syst Rev* 2018;4:CD005951.
9. Keeling D, Tait C, Makris M. Guideline on the selection and use of therapeutic products to treat haemophilia and other hereditary bleeding disorders. *Br J Haematol* 2008;14:671-84.
10. Laffan MA, Lester W, O'Donnell JS, et al. The diagnosis and management of von Willebrand disease. *Br J Haematol* 2014;167:453-65.
11. Estcourt LJ, Birchall J, Allard S, et al. Guidelines for the use of platelet transfusions. *Br J Haematol* 2017;176:365-94.

For peer review only

12. Schneider SW, Nuschele S, Wixforth A, et al. Shear-induced unfolding triggers adhesion of Von Willebrand Factor fibers. *Proc Natl Acad Sci U S A* 2007;104:7899-903
13. Colucci G, Stutz M, Rochat S, et al. The effect of desmopressin on platelet function: a selective enhancement of procoagulant COAT platelets in patients with primary platelet function defects. *Blood* 2014;123:1905-16.
14. Frontera JA, Lewin JJ 3rd, Rabinstein AA, et al. Guideline for reversal of antithrombotics in intracranial hemorrhage: a statement for healthcare professionals from the Neurocritical Care Society and Society of Critical Care Medicine. *Neurocrit Care* 2016;24:6-46.
15. Intercollegiate stroke working party. National clinical guideline for stroke. London, UK: Royal College of Physicians 2016.
16. Desborough MJ, Oakland KA, Landoni G, et al. Desmopressin for treatment of platelet dysfunction and reversal of antiplatelet agents: a systematic review and meta-analysis of randomized controlled trials. *J Thromb Haemost* 2017;15:263-72.
17. Desborough MJ, Oakland K, Brierley C, et al. Desmopressin for minimising perioperative blood transfusion. *Cochrane Database Syst Rev* 2017;7:CD001884
18. Naidech AM, Maas MB, Levasseur-Franklin KE, et al. Desmopressin improves platelet activity in acute intracerebral hemorrhage. *Stroke* 2014;45:2451-3.
19. Kapapa T, Rohrer S, Struve S, et al. Desmopressin acetate in intracranial haemorrhage. *Neurol Res Int* 2014;2014:298767.
20. Feldman EA, Meola G, Zyck S, et al. Retrospective assessment of desmopressin effectiveness and safety in patients with antiplatelet-associated intracerebral hemorrhage. *Crit Care Med* 2019;47:1759-65.
21. Schmidt KJ, Sager B, Zachariah J, et al. Cohort analysis of desmopressin effect on haematoma expansion in patients with spontaneous intracerebral hemorrhage and documented pre-ictus antiplatelet use. *J Clin Neurosci* 2019;66:33-7.
22. Weir CJ, Lees KR. Comparison of stratification and adaptive methods for treatment allocation in an acute stroke clinical trial. *Stat Med* 2003;22:705-26.
23. Bath PM, May J, Flaherty K, et al. Remote Assessment of Platelet Function in Patients with Acute Stroke or Transient Ischaemic Attack. *Stroke Res Treat* 2017;2017:7365684.

For peer review only

24. Moher D, Hopewell S, Schulz KF, et al. CONSORT 2010 explanation and elaboration: updated guidelines for reporting parallel group randomised trials. *BMJ* 2010;340:c869.
25. Schulz KF, Altman DG, Moher D. CONSORT 2010 statement: updated guidelines for reporting parallel group randomised trials. *BMJ* 2010;340:c332.
26. Sprigg N, Flaherty K, Appleton JP, et al. Tranexamic acid for hyperacute primary IntraCerebral Haemorrhage (TICH): an international randomised, placebo-controlled, phase 3 superiority trial. *Lancet* 2018;391:2107-15.
27. Baharoglu MI, Cordonnier C, Al-Shahi Salman R, et al. Platelet transfusion versus standard care after acute stroke due to spontaneous cerebral haemorrhage associated with antiplatelet therapy (PATCH): a randomised, open- label, phase 3 trial. *Lancet* 2016;387:2605-13.

Authors' contributions: MD, SS, RS and NS conceived the ideas for the study. MD wrote the first draft of the protocol with RS, SS and NS. DH wrote the sections on trial regulation. TH wrote the statistical analysis plan. PB, RD, TC, PJ and PB critically reviewed the protocol and provided expert input.

Funding statement: This paper presents independent research funded by the National Institute for Health Research (NIHR) under its Research for Patient Benefit (RfPB) Programme (Grant Reference Number PB-PG-0816-20011). The views expressed are those of the author(s) and not necessarily those of the NIHR or the Department of Health and Social Care

Competing interests statement: The authors have no competing interests to declare.

Word Count: 3471 (up to 4,000 words).

For peer review only

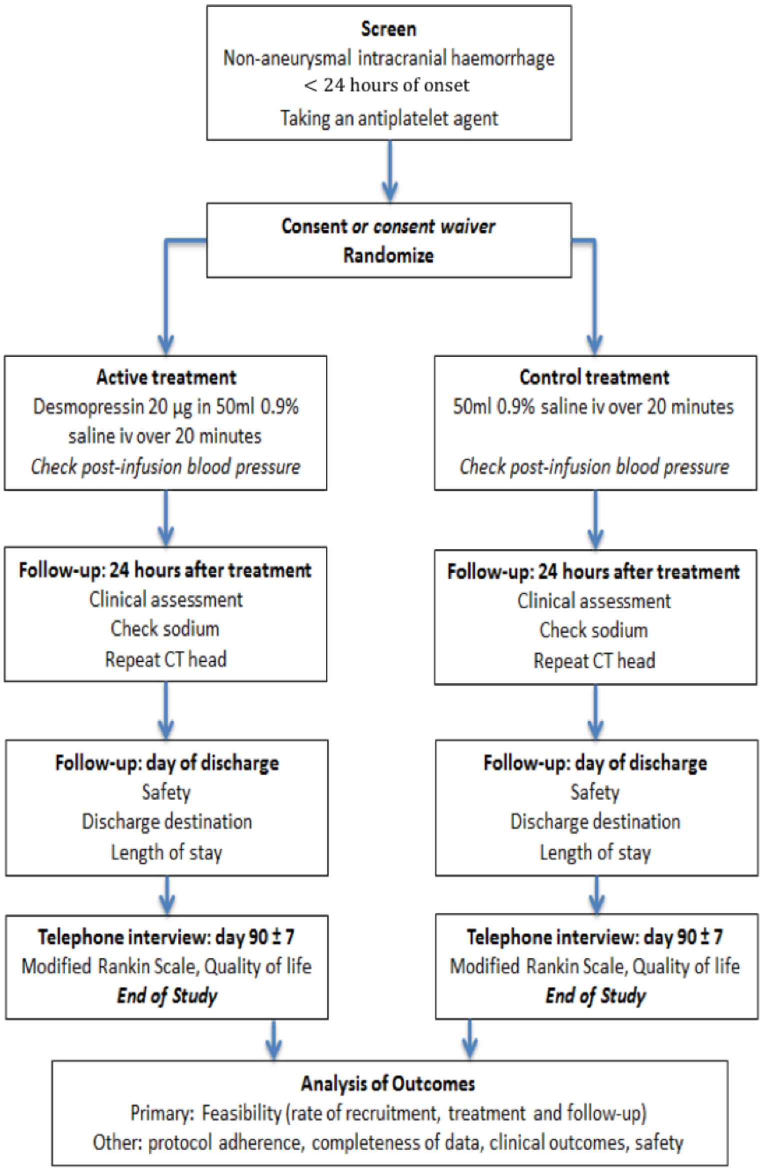

Figure 1. Summary of patient flow through the trial.

BMJ Open

Desmopressin for reversal of Antiplatelet drugs in Stroke due to Haemorrhage (DASH): protocol for a phase II double blind randomised controlled feasibility trial

Journal:	*BMJ Open*
Manuscript ID	bmjopen-2020-037555.R1
Article Type:	Protocol
Date Submitted by the Author:	09-Jun-2020
Complete List of Authors:	Desborough, Michael; Guy's and Saint Thomas' Hospitals NHS Trust, Haemostasis and Thrombosis Centre<br>Salman, Rustam; University of Edinburgh Western General Hospital, Centre for Clinical Brain Sciences<br>Stanworth, Simon; Oxford University Hospitals NHS Foundation Trust, Department of Haematology; University of Oxford, NIHR Oxford Biomedical Research Centre, Radcliffe Department of Medicine<br>Havard, Diane; University of Nottingham, Division of Clinical Neuroscience<br>Brennan, Paul; University of Edinburgh Western General Hospital, Centre for Clinical Brain Sciences<br>Dineen, Rob; University of Nottingham, Division of Clinical Neuroscience; University of Nottingham, NIHR Nottingham Biomedical Research Centre<br>Coats, Timothy; University of Leicester, Department of Cardiovascular Sciences<br>Hepburn, Trish; University of Nottingham, Clinical Trials Unit<br>Bath, Philip; University of Nottingham, Division of Clinical Neuroscience<br>Sprigg, Nikola; University of Nottingham, Division of Clinical Neuroscience
**Primary Subject Heading**:	Neurology
Secondary Subject Heading:	Emergency medicine, Evidence based practice, Haematology (incl blood transfusion), Neurology
Keywords:	Stroke medicine < INTERNAL MEDICINE, Bleeding disorders & coagulopathies < HAEMATOLOGY, Anticoagulation < HAEMATOLOGY, Stroke < NEUROLOGY, Clinical trials < THERAPEUTICS

SCHOLARONE™
Manuscripts

I, the Submitting Author has the right to grant and does grant on behalf of all authors of the Work (as defined in the below author licence), an exclusive licence and/or a non-exclusive licence for contributions from authors who are: i) UK Crown employees; ii) where BMJ has agreed a CC-BY licence shall apply, and/or iii) in accordance with the terms applicable for US Federal Government officers or employees acting as part of their official duties; on a worldwide, perpetual, irrevocable, royalty-free basis to BMJ Publishing Group Ltd ("BMJ") its licensees and where the relevant Journal is co-owned by BMJ to the co-owners of the Journal, to publish the Work in this journal and any other BMJ products and to exploit all rights, as set out in our licence.

The Submitting Author accepts and understands that any supply made under these terms is made by BMJ to the Submitting Author unless you are acting as an employee on behalf of your employer or a postgraduate student of an affiliated institution which is paying any applicable article publishing charge ("APC") for Open Access articles. Where the Submitting Author wishes to make the Work available on an Open Access basis (and intends to pay the relevant APC), the terms of reuse of such Open Access shall be governed by a Creative Commons licence – details of these licences and which Creative Commons licence will apply to this Work are set out in our licence referred to above.

Other than as permitted in any relevant BMJ Author's Self Archiving Policies, I confirm this Work has not been accepted for publication elsewhere, is not being considered for publication elsewhere and does not duplicate material already published. I confirm all authors consent to publication of this Work and authorise the granting of this licence.

For peer review only

Long title: Desmopressin for reversal of Antiplatelet drugs in Stroke due to Haemorrhage (DASH): protocol for a phase II double blind randomised controlled feasibility trial

Short title: DASH trial

Authors: Michael J R Desborough[1], Rustam Al-Shahi Salman[2], Simon J Stanworth[3,4,5], Diane Havard[6], Paul M Brennan[2], Robert A Dineen[6], Tim J Coats[7], Trish Hepburn[8], Philip M Bath[6], Nikola Sprigg[6]; DASH trial investigators

[1]Haemostasis and Thrombosis Centre, St Thomas' Hospital, London, [2]Centre for Clinical Brain Sciences, University of Edinburgh, Edinburgh, [3]Transfusion Medicine, NHS Blood and Transplant, Oxford, [4]Department of Haematology, Oxford University Hospitals NHS Foundation Trust, [5]Radcliffe Department of Medicine, University of Oxford, and NIHR Oxford Biomedical Research Centre, [6]Division of Clinical Neuroscience, University of Nottingham, Nottingham and Nottingham Biomedical Research Centre [7]Department of Cardiovascular Sciences, University of Leicester, Leicester, [8]Clinical Trials Unit, University of Nottingham, Nottingham, United Kingdom

Corresponding Author:

Professor Nikola Sprigg

Professor of Stroke Medicine

Room B60 Clinical Sciences Building

Nottingham City Hospital

Hucknall Road

Nottingham

NG5 1PB

United Kingdom

Phone +44 (0) 115 823 1778

Fax +44 (0) 115 823 1767

Email: nikola.sprigg@nottingham.ac.uk

For peer review only

Abstract

Introduction

Intracerebral haemorrhage can be devastating and is a common cause of death and disability worldwide. Pre-intracerebral haemorrhage antiplatelet drug use is associated with a 27% relative increase in one-month case fatality compared to patients not using antithrombotic drugs. We aim to assess the feasibility of conducting a randomised controlled testing the safety and efficacy of desmopressin for patients with antiplatelet-associated intracerebral haemorrhage.

Methods and Analysis

We aim to include 50 patients within 24 hours of spontaneous intracerebral haemorrhage onset, associated with oral antiplatelet drug(s) use in at least the preceding seven days. Patients will be randomised (1:1) to receive intravenous desmopressin 20μg in 50 mls sodium chloride 0.9% infused over 20 minutes or matching placebo. We will mask participants, relatives and outcome assessors to treatment allocation. Feasibility outcomes include proportion of patients approached being randomised, number of patients receiving allocated treatment, rate of recruitment, and adherence to treatment and follow up. Secondary outcomes include change in intracerebral haemorrhage volume at 24 hours; hyponatraemia at 24 hours, length of hospital stay, discharge destination, early death less than 28 days, death or dependency at day 90, death up to day 90, serious adverse events (including thromboembolic events) up to day 90; disability (Barthel index, day 90), quality of life (EuroQol 5D (EQ-5D], day 90), cognition (telephone mini-mental state examination day 90), and health economic assessment (EQ-5D).

Ethics and dissemination

The DASH trial received ethical approval from the East Midlands - Nottingham 2 research ethics committee (18/EM/0184). The DASH trial is funded by NIHR RfPB grant: PB-PG-0816-20011. Trial results will be published in a peer reviewed academic journal and disseminated through academic conferences and through patient stroke support groups. Reporting will be in compliance with CONSORT recommendations.

For peer review only - http://bmjopen.bmj.com/site/about/guidelines.xhtml

Trial Registration: NCT03696121; ISRCTN67038373; EudraCT 2018-001904-12

Protocol version number: Version 2.0; 26th August 2019

Strengths and limitations of the study

- This is the first randomised controlled trial of desmopressin versus placebo for patients taking antiplatelet drugs with spontaneous intracerebral haemorrhage
- Robust methodology developed from large international multicentre trials in spontaneous intracerebral haemorrhage such as TICH-2 and PATCH
- Desmopressin and placebo are not identical so the person administering the intervention will not be masked to treatment allocation.
- All outcomes assessors are blinded to treatment allocation to minimize the risk of bias

For peer review only

For peer review only - http://bmjopen.bmj.com/site/about/guidelines.xhtml

Introduction

Haemorrhagic stroke

Spontaneous intracerebral haemorrhage (ICH) causes approximately 3 million deaths per year worldwide [1] and is responsible for the loss of 64.5 million disability adjusted life years per year [2]. Two-thirds of survivors are left dependent on others[3]. Roughly one third of patients are taking antiplatelet drugs at the time of ICH in high-income countries, and this proportion has been increasing over time[4]. Pre-ICH antiplatelet drug use is associated with a 27% relative increase in one-month case fatality compared to patients not using antithrombotic drugs[5]. Despite development of effective treatments for ischaemic stroke (thrombolysis, thrombectomy, aspirin, hemicraniectomy) there is no proven effective drug treatment for ICH, although blood pressure lowering might improve outcome[6].

Haematoma expansion

Outcome after ICH is closely related to haematoma growth (expansion) which is associated with worse outcome (death and disability)[7]. Use of antiplatelet drugs or anticoagulants, time from onset of symptoms to baseline imaging, and intracerebral haemorrhage volume on baseline imaging are independent predictors of haematoma expansion[8].

Platelet transfusion, tranexamic acid and recombinant activated factor VII have not been shown to improve outcomes after anti-platelet associated intracerebral haemorrhage, raising the need to consider alternatives [9].

Desmopressin

Desmopressin is a licensed pro-haemostatic drug that can be administered intravenously, subcutaneously, intranasally or orally and is used in a number of inherited bleeding conditions to treat or prevent bleeding[10-12]. Platelet function remains inhibited for 5-7 days after stopping antiplatelet drugs. Desmopressin stimulates release of Von Willebrand Factor (VWF) and factor VIII from endothelial Weibel-Palade bodies. VWF is responsible for platelet adhesion to collagen and may also bind platelets through their glycoprotein IIb/IIIa receptors [13], so increased

For peer review only

levels of VWF have the potential to compensate for the platelet function defect associated with antiplatelet drugs. Desmopressin may also increase the formation of procoagulant platelets [14].

Desmopressin is recommended for the reversal of antiplatelet drugs in guidelines from the USA[15] but not in UK guidelines[16]. In a recent meta-analysis evaluating desmopressin for reversal of antiplatelet drugs for patients undergoing cardiac surgery, desmopressin was found to reduce blood loss, transfusion requirements and the need for a further operation due to bleeding[17].

There are potential risks with use of desmopressin, including tachycardia hypotension, hyponatraemia and hyponatraemic seizures. A meta-analysis of 65 surgical randomised controlled trials, comparing desmopressin to placebo found no significant difference in rates of ischaemic stroke, myocardial infarction or venous thromboembolism[18].

Desmopressin has been tested in ICH in four case series where it appears to be safe[19-22]. However the series were small, non-randomised and did not have a placebo control arm, so it is not possible to make a clear assessment of the benefits and harms of administering desmopressin for these patients. American neurocritical care guidelines for the treatment of intracranial haemorrhage recommend consideration of 0.4 mcg/kg desmopressin for treatment of antiplatelet-associated ICH [15], although the level of evidence supporting this recommendation was recognised as very uncertain.

Searches of clinicaltrials.gov and World Health Organization International Trials Clinical Registry Platform found no other ongoing randomised controlled trials of desmopressin versus placebo for reversal of anti-platelet drugs in stroke due to haemorrhage.

If it is feasible to recruit patients and to collect robust data then we intend to proceed to a definitive trial to test efficacy.

For peer review only - http://bmjopen.bmj.com/site/about/guidelines.xhtml

Primary Objective

To assess the feasibility of randomising, administering the intervention, and completing follow-up for patients treated with desmopressin or placebo to inform a definitive trial.

Methods and analysis

DASH will be a multicentre double-blind randomised placebo-controlled, parallel group phase II feasibility trial. Participants will be enrolled by investigators from emergency departments or acute stroke units from 10 hospital sites in the United Kingdom. Patient flow though the trial is summarised in Figure 1.

Inclusion criteria:

- Adults (≥18 years)
- Confirmed ICH on imaging
- Randomised less than 24 hours from onset of symptoms [or from when last seen free of stroke symptoms]
- Prescribed and thought to be taking a daily oral antiplatelet drug in the preceding seven days (cyclooxygenase inhibitors, phosphodiesterase inhibitors or $P2Y_{12}$ inhibitors).

Exclusion criteria:

- Known secondary causes of ICH (aneurysmal subarachnoid haemorrhage or haemorrhage due to transformation of infarction, thrombolytic drug, venous thrombosis, arteriovenous malformation or tumour)
- Patients at risk of fluid retention
- Systolic blood pressure less than 90mmHg
- Known drug eluting stent in previous 3 months
- Allergy to desmopressin
- Pregnant or breast-feeding
- Life-expectancy less than 4 hours or planned for palliative care only
- Glasgow coma scale less than 5

For peer review only - http://bmjopen.bmj.com/site/about/guidelines.xhtml

- Modified Rankin scale (mRS) more than 4.

Consent:

Patients with capacity to give consent will be approached directly. If this is not possible, a relative or close friend will be approached for consent, if this is not possible then a professional not associated with the trial will be approached for proxy consent. Patients will be approached again for consent when they regain capacity (supplementary files 1-3; all trial documents are available at: http://dash-1.ac.uk/docs/public.php).

Randomisation:

Participants will be randomised centrally using a secure internet site in real-time. Treatment allocation will be concealed from all staff and patients involved in the trial. Randomisation involves minimisation on key prognostic risk factors: age (70 years or more); sex (male); time since onset (3 hours or more); systolic blood pressure (170mmHg or more); and presence (or no information on presence or absence) of intraventricular haemorrhage. This approach ensures concealment of allocation, minimises differences in key baseline prognostic variables, and slightly improves statistical power [23]. Randomisation will allocate a number corresponding to a treatment pack and the participant will receive treatment from the allocated numbered pack. It was considered not possible to mask/blind personnel preparing the injection for administration to the treatment allocation because the ampoules of active and placebo injections are different and carry the manufacturer's identifying information. Clinicians, patients and outcome assessors (clinical, radiological and haematological assessors) will be blinded to treatment allocation.

In general, there should be no need to unblind the allocated treatment. If some contraindication to desmopressin develops after randomisation, the trial treatment should be stopped. Unblinding should be done only in those rare cases when the doctor believes that clinical management depends importantly upon knowledge of whether the patient received desmopressin or placebo. In those few cases when urgent unblinding is considered necessary, the doctor will call an emergency

For peer review only

telephone number. The caller will then be told whether the patient received desmopressin or placebo. The rate of unblinding will be monitored and audited.

Intervention:

Patients will be randomised to either 20 µg intravenous desmopressin (DDAVP, 4µg/ml 1ml glass ampoules, Ferring Pharmaceuticals) or placebo of intravenous Sodium Chloride 0.9%. Placebo was selected for comparison because at present there are no established haemostatic therapies for ICH. Either desmopressin 20µg (5x 4µg/ml) will be added to 50ml Sodium Chloride 0.9% and infused over 20 minutes, or three 2ml Sodium Chloride 0.9% will be added to 50ml Sodium Chloride 0.9% and infused over 20 minutes. The investigational medicinal product (IMP) will be stored between 2◦C and 8◦C until use. All patients will receive all usual standard care including venous thromboembolism prophylaxis with intermittent pneumatic compression devices, blood pressure lowering therapy and neurosurgery if appropriate.

We will administer a single dose as the risk of continued bleeding and haematoma expansion after ICH is greatest in the first few hours. High levels of Factor VIII and VWF are maintained for 6–8 h after intravenous infusion therefore we believe a repeated dose at 12-24 hours would not be indicated. This dose regime (0.4 micrograms per kilogram body weight administered by intravenous infusion) is recommended in American neurocritical care guidelines for the treatment of intracranial haemorrhage[15], although the level of evidence supporting this recommendation was recognised as very uncertain .

Assessments:

Participants' age, sex, medical history, antiplatelet drug use, intracerebral haemorrhage location, intraventricular haemorrhage and mRS will be assessed at baseline by local investigators. Participants will be reviewed again on day 2, hospital discharge and on day 90 to gather information on interventions, adverse events, discharge date and discharge destination. Data collection forms can be found at: https://dash-1.ac.uk/

For peer review only - http://bmjopen.bmj.com/site/about/guidelines.xhtml

At day 90 central assessors trained in the appropriate questionnaires and masked to treatment allocation will follow up each patient by telephone. Barthel index, mRS, EuroQOL, cognition (mini-mental state examination) and serious adverse events will be assessed by telephone.

Brain imaging (CT head) will be undertaken as part of routine care before enrolment. A second research CT head scan will be performed 24 hours after treatment with the IMP to measure parenchymal haematoma volume. Haematoma volume will be measured centrally by trained assessors masked to treatment allocation using semi-automated segmentation. We will assess change in haematoma volume at 24 hours, and proportion of patients with haematoma expansion, defined as an absolute increase of greater than 6ml or relative growth of greater than 33%.

To assess baseline platelet function, platelets will be stimulated with arachidonic acid (which is inhibited by aspirin) or adenosine diphosphate (which is inhibited by P2Y12 inhibitors). Platelet cell surface P-selectin expression (a measure of platelet activation) will then be measured using a standardised assay [Heptinstall; patent pending (PTC/GB2008/050169)] to assess retrospectively whether patients were taking antiplatelet drugs, as previously described [24]. Von Willebrand factor (VWF) antigen, VWF activity and factor VIII (1-stage) will be measured centrally by assessors masked to treatment allocation. Blood samples will be drawn into 0.109M citrate and centrifuged at 2500g for 15 minutes. Platelet poor plasma will be aspirated and stored at -80◦C at each site and then transferred to the central laboratory at the end of the trial for analysis. These tests will be performed for research only and the results will not be fed back to the clinical team. Serum sodium will be assessed locally 24 hours after administration of the IMP and these results will be available to the clinical team.

Outcomes:

This is a feasibility trial and the primary outcomes are based around feasibility: Number of eligible patients who receive allocated treatment; rate of eligible patients randomised; proportion of eligible patients approached; proportion of eligible patients randomised and reasons for non-randomisation; adherence to intervention; proportion of participants followed up to 90 days and reasons for loss to follow up;

For peer review only - http://bmjopen.bmj.com/site/about/guidelines.xhtml

proportion of randomised participants with full outcome data available, and reasons for non-availability.

Secondary outcomes data will be collected to inform the design of a definitive trial but will not be statistically analysed. Secondary outcomes are change in intracerebral haemorrhage volume at 24 hours; hyponatraemia at 24 hours, length of hospital stay, discharge destination, early mortality less than 28 days, death or dependency at day 90, mortality up to day 90, serious adverse events (including thromboembolic events) up to day 90; disability (Barthel index, day 90), quality of life (EuroQol, day 90), cognition (telephone MMSE day 90), and health economic assessment (EQ-5D). Baseline platelet dysfunction will be measured and correlated with response to desmopressin; and change in factor VIII, VWF antigen and VWF activity will be assessed one hour after administration of desmopressin.

Adverse events:

All adverse events on Day 1 (including during infusion) and for the 24-hour period post dose will be collected. All adverse events will be assessed for seriousness, expectedness and causality by adjudicators masked to treatment allocation. Serious adverse events will be categorised in accordance with the medical dictionary for regulatory authorities (MeDRA). As the IMP is administered once and has a short half-life, serious adverse events occurring within the first 7 days will be assessed for seriousness, expectedness and causality. In addition, fatal SAEs and safety outcome events (fluid overload, hyponatraemia) will be reported until day 90.

Statistical analysis

Data will be analysed by a qualified statistician who is blinded to treatment allocation, using a validated software package. A statistical analysis plan (SAP) will be agreed prior to database lock and release of randomisation codes. The trial will be reported in accordance with CONSORT guidelines including the extension to pilot and feasibility trials, as appropriate [25-26]. This is a feasibility trial and the main analysis will be with descriptive statistics only. Counts will be summarised using N and %, and continuous variables will be summarised using means and standard deviations or medians and interquartile ranges depending on their distribution. Whilst some

For peer review only

variables will be summarised by treatment group, no formal statistical comparisons will be made and any analyses will be considered purely exploratory. We will assess the feasibility of recruiting, treating and following up patients from 10 centres over one year. We will estimate a recruitment rate, treatment rate and follow-up rate. It is likely that a large definitive trial would be feasible if at least 50 participants were recruited into this study, that compliance with randomised treatment was high and that a high proportion of follow up data was available. Lower recruitment would not preclude progression if there was some evidence that the barriers to recruitment identified could be overcome. A decision about feasibility for a larger trial will be made in conjunction with the trial steering committee taking into account recruitment rates, differences between centres and other relevant information.

This is a feasibility trial and as such will have no formal assessment of efficacy. The proposed primary efficacy outcome in a definitive trial would be death or dependency at day 90, measured using the mRS. Shifts in the mRS will be summarised for this trial but no formal confirmatory statistical analyses will be performed. Similarly, all other efficacy variables will be summarised using descriptive statistics. Serious adverse events will be summarised using descriptive statistics according to the treatment the participant received. Missing data will be reported. The investigation of this data and methods implemented to address the missing data, if appropriate, will be detailed in the SAP.

All available data will be used including overall numbers of patients presenting in clinic and screening data (where available). Where summaries by treatment group are provided, these will be based on an intention to treat population i.e. according to the treatment the participant was randomised to, with the exception of safety data. A safety population will be defined to summarise the safety data in this study where participants will be summarised according to the treatment they received irrespective of randomisation. Summaries of the number and proportion of participants who would form a per protocol population in a larger trial, and reasons for exclusion from a per protocol population will be provided to allow future planning. No other data will be summarised for this population.

For peer review only - http://bmjopen.bmj.com/site/about/guidelines.xhtml

Sample size and justification

Since this is a feasibility study with one of the objectives being to determine potential recruitment rates, a formal sample size calculation is not appropriate. If more than 50 participants were randomised from 10 centres in a 12 month period, it is likely that, assuming similar recruitment rates in additional centres, a larger study recruiting approximately 1200 participants in around 50 centres recruiting for 60 months would be feasible. Depending on the final sample size calculation for the definitive study, the number of centres and recruitment period could be determined using the information from the rates and patterns observed in the feasibility study. Information about set up times for centres will also better inform recruitment projections for a larger study. TICH-2 and other studies indicate approximately 25% of all people presenting with ICH are taking antiplatelet drugs [27].

Patient and public involvement

This study was developed in collaboration with, and is supported by Nottingham Stroke Research Partnership Group, made up of stroke survivors and carers. Members of the group have reviewed the proposed study, and in an iterative process, commented on its design and conduct. In particular, the group has influenced the approach to taking informed consent within the study. In addition, the proposal was also reviewed by the Research to Understand Stroke due to Haemorrhage (RUSH) patient reference group in Edinburgh (http://www.ed.ac.uk/clinical-brain-sciences/research/diagnoses-diseasetargets/rush/rush-patient-reference-group).

Ethics and dissemination

The trial will be overseen by a trial steering committee. A trial management committee based at the Stroke Trials Unit in Nottingham, UK will be responsible for day-to-day conduct of the trial. An independent data monitoring committee will review the data at six months. Study data will be collected, monitored and analysed in Nottingham. The data management policy is available at: http://dash-1.ac.uk/docs/public.php. The trial will be run in accordance with the principles of good clinical practice and the Declaration of Helsinki. The DASH trial has been granted ethics approval by the East Midlands - Nottingham 2 research ethics committee (18/EM/0184). The trial has been adopted in the UK by the National Institute for

For peer review only - http://bmjopen.bmj.com/site/about/guidelines.xhtml

Health and Care Research (NIHR) clinical trial network and is registered as NCT03696121, ISRCTN67038373 and EudraCT 2018-001904-12.

Trial results will be published in a peer reviewed academic journal. Reporting will be in compliance with CONSORT[25-26] recommendations. When the study is complete summary findings will be posted on the patient support group website. Findings will also be presented at conferences such as the UK Stroke Forum, European Stroke Conference, World Stroke Congress, British Society for Haematology annual meeting, and International Society on Thrombosis and Haemostasis annual meeting.

Protocol amendments

Should a protocol amendment be made that requires ethics approval, the changes in the protocol will not be instituted until the amendment and revised informed consent forms and participant information sheets have been reviewed and received approval/favourable opinion from the research ethics committee and research and development departments. The results will be communicated to all principal investigators.

In the initial version of the protocol, which was in place from the start of the trial until 30/11/2019, participants could be recruited if they could be randomised less than 12 hours from onset of symptoms [or from when last seen free of stroke symptoms]. In the updated version of the protocol, which came into effect on 01/12/2019, participants could be recruited if they could be randomised less than 24 hours from onset of symptoms [or from when last seen free of stroke symptoms].

Confidentiality and Access to Data

All trial staff and investigators will endeavour to protect the rights of the trial's participants to privacy and informed consent, and will adhere to the Data Protection Act, 2018. The case report forms will only collect the minimum required information for the purposes of the trial. Case report forms will be held securely, in a locked room, or locked cupboard or cabinet. Access to the information will be limited to the trial staff and investigators and relevant regulatory authorities. Computer held data including the trial database will be held securely and password protected. All data will

For peer review only - http://bmjopen.bmj.com/site/about/guidelines.xhtml

be stored on a secure dedicated web server. Access will be restricted by user identifiers and passwords. Information about the trial in the participant's medical records/hospital notes will be treated confidentially in the same way as all other confidential medical information. Electronic data will be backed up every 24 hours to both local and remote media in encrypted format.

Insurance and Indemnity

Insurance and indemnity for trial participants and trial staff is covered within the NHS Indemnity Arrangements for clinical negligence claims in the NHS, issued under cover of HSG (96)48. There are no special compensation arrangements, but trial participants may have recourse through the NHS complaints procedures. The University of Nottingham as research Sponsor indemnifies its staff, research participants and research protocols with both public liability insurance and clinical trials insurance. These policies include provision for indemnity in the event of a successful litigious claim for proven non-negligent harm.

Discussion

This will be the first randomised controlled trial comparing desmopressin to placebo for patients with ICH who are taking antiplatelet drugs. ICH is a common cause of death and disability worldwide. Patients taking antiplatelet drugs are more likely to have haematoma expansion and to have poorer outcomes than those who are not taking an antiplatelet drug. Early treatment with drugs to reduce the effect of antiplatelet drugs may reduce haematoma expansion. Desmopressin is a promising drug, which is commonly used in the treatment of inherited bleeding disorders and may reduce the effects of antiplatelet drugs.

In this trial we aim to determine if it is feasible to administer desmopressin within 24 hours to patients with ICH taking an antiplatelet drug. One potential barrier is that some patients will have delayed presentations to hospital. For those that reach hospital within 24 hours, we have aimed to make recruitment to the trial streamlined by minimising barriers to recruitment. This includes use of broad inclusion criteria and using a simple trial design. The design of this trial is based on the successful TICH-2[27] and PATCH[28] multicentre trials. The use of consent from a relative, close

For peer review only - http://bmjopen.bmj.com/site/about/guidelines.xhtml

friend or professional representative is essential in this trial because the majority of patients will lack capacity.

If it proves feasible to run a trial of desmopressin compared to placebo for these patients then we aim to proceed to a large efficacy study. This will have an important impact on clinical practice. Desmopressin is widely available and inexpensive. It has the potential to reduce the risk of death and disability for patients with ICH who are taking antiplatelet drugs. Desmopressin could be rapidly adopted into clinical guidelines if it proves to reduce the risk of death or disability for patients with ICH.

Funding statement: This paper presents independent research funded by the National Institute for Health Research (NIHR) under its Research for Patient Benefit (RfPB) Programme (Grant Reference Number PB-PG-0816-20011). The views expressed are those of the author(s) and not necessarily those of the NIHR or the Department of Health and Social Care. The trial sponsor and funders have no role in the; collection, management, analysis, and interpretation of data; writing of the report; and the decision to submit the report for publication

Trial sponsor contact: Ms Angela Shone, Head of Research Governance, Research and Innovation, University of Nottingham, East Atrium, Jubilee Conference Centre, Triumph Road, Nottingham, NG8 1DH

Trial Management Group

The Trial Management Group (TMG) will meet regularly, at least every four weeks to run the trial. TMG members are: Nikola Sprigg - Chief Investigator, Michael Desborough - Deputy Chief Investigator, Diane Havard - Senior Clinical Trials Manager, Sharon Ellender - Trial Coordinator, Lee Haywood – Programmer, Lisa Woodhouse - Trial statistician, and Patricia Robinson - Trial Administrator

For peer review only

Trial Steering Committee

The independent Trial Steering Committee (TSC) will provide oversight of the trial. It will meet (in person or by telephone conference) prior to commencement of the trial, and then at regular intervals until completion (at least annually). Specific tasks of the TSC are:

- to approve the trial protocol
- to approve necessary changes to the protocol based on considerations of feasibility and practicability
- to receive reports from the Data Safety Monitoring Committee
- to resolve problems brought to it by the co-ordinating centre and TMG
- to ensure publication of the trial results
- to advise on whether the main phase of the trial is feasible

TSC members are: Colin Baigent – Chair, Christine Knott – patient and public involvement representative, Mathew Walters - patient and public involvement representative, Angela Shone – Sponsor, Trish Hepburn – Statistician, Philip Bath - Prof of Stroke Medicine, Rob Dineen - Prof of Neuro-radiology, Paul Brennan – neurosurgeon, Rustam Al-Shahi Salman - Prof of Stroke Medicine, Laura Green - Senior Clinical Lecturer in Transfusion Research & Innovation, Simon Stanworth – Associate Professor of Haematology, Tim Coates - Prof of Emergency Medicine, Phil Johnson - patient and public involvement representative, Emily Toon - Funder representative

The Trial Coordinator, or where required, a nominated designee of the Sponsor, shall carry out a site systems audit at least yearly and an audit report shall be made to the Trial Steering Committee.

Data Monitoring Committee

The independent Data Monitoring Committee (DMC) will receive safety reports every six months, or more frequently if requested and perform unblinded reviews of safety data. The DMC will report their assessment to the independent chair of the TSC. Collaborators, and all others associated with the trial, may write through the trial office to the DMC, to draw attention to any concern they may have about the trial interventions, or any other

For peer review only

relevant issues. There will not be an interim analysis. DMC members are: John Bamford – Chair, Graham Venables - Prof of Neurology, Martin Bland - Prof of Statistics

Participating United Kingdom Centres: Nottingham University Hospitals NHS Trust; NHS Grampian; University College London Hospitals NHS Foundation Trust; NHS Lothian; Royal Devon & Exeter NHS Foundation Trust; University Hospitals of Leicester NHS Trust; University Hospitals of North Midlands NHS Trust; Derby Teaching Hospitals NHS Foundation Trust; Newcastle Upon Tyne Hospitals NHS Foundation Trust; St George's University Hospitals NHS Foundation Trust

Authors' contributions: MD, SS, RS and NS conceived the ideas for the study. MD wrote the first draft of the protocol with RS, SS and NS. DH wrote the sections on trial regulation. TH wrote the statistical analysis plan. PB, RD, TC, PJ and PB critically reviewed the protocol and provided expert input.

Competing interests statement: The authors have no competing interests to declare.

Figure 1: Study Flowchart

Word Count: 3990

References

1. GBD 2015 Mortality and Causes of Death Collaborators. Global, regional, and national life expectancy, all-cause mortality, and cause-specific mortality for 249 causes of death, 1980-2015: a systematic analysis for the Global Burden of Disease Study 2015. *Lancet* 2016;388:1459-544.
2. GBD 2017 DALYs and HALE collaborators. Global, regional, and national disability-adjusted life-years (DALYs) for 359 diseases and injuries and healthy life-expectancy (HALE) for 195 countries and territories, 1990-2017: a systematic analysis for the Global Burden of Disease Study 2017. *Lancet* 2018;392:1859-922
3. Royal College of Physicians. Sentinal Stroke National Audit Programme (SSNAP). 2016.

For peer review only

4. Lovelock CE, Molyneux AJ, Rothwell PM. Change in incidence and aetiology of intracerebral haemorrhage in Oxfordshire, UK, between 1981 and 2006: a population-based study. *Lancet Neurol* 2007;6:487-93.
5. Thompson BB, Bejot Y, Caso V, et al. Prior antiplatelet therapy and outcome following intracerebral hemorrhage: a systematic review. *Neurology* 2010;75:1333-42.
6. Anderson CS, Heeley E, Huang Y, et al. Rapid blood-pressure lowering in patients with acute intracerebral hemorrhage. *N Engl J Med* 2013;368:2355-65.
7. Steiner T, Bosel J. Options to restrict hematoma expansion after spontaneous intracerebral hemorrhage. *Stroke* 2010;41:402-9.
8. Al-Shahi Salman R, Frantzias J, Lee RJ, et al. Absolute risk and predictors of the growth of acute spontaneous intracerebral haemorrhage: a systematic review and meta-analysis of individual patient data. *Lancet Neurol* 2018;17:885-94
9. Al-Shahi Salman R, Law ZK, Bath PM, et al. Haemostatic therapies for acute spontaneous intracerebral haemorrhage. *Cochrane Database Syst Rev* 2018;4:CD005951.
10. Keeling D, Tait C, Makris M. Guideline on the selection and use of therapeutic products to treat haemophilia and other hereditary bleeding disorders. *Br J Haematol* 2008;14:671-84.
11. Laffan MA, Lester W, O'Donnell JS, et al. The diagnosis and management of von Willebrand disease. *Br J Haematol* 2014;167:453-65.
12. Estcourt LJ, Birchall J, Allard S, et al. Guidelines for the use of platelet transfusions. *Br J Haematol* 2017;176:365-94.
13. Schneider SW, Nuschele S, Wixforth A, et al. Shear-induced unfolding triggers adhesion of Von Willebrand Factor fibers. *Proc Natl Acad Sci U S A* 2007;104:7899-903
14. Colucci G, Stutz M, Rochat S, et al. The effect of desmopressin on platelet function: a selective enhancement of procoagulant COAT platelets in patients with primary platelet function defects. *Blood* 2014;123:1905-16.
15. Frontera JA, Lewin JJ 3rd, Rabinstein AA, et al. Guideline for reversal of antithrombotics in intracranial hemorrhage: a statement for healthcare

For peer review only - http://bmjopen.bmj.com/site/about/guidelines.xhtml

professionals from the Neurocritical Care Society and Society of Critical Care Medicine. *Neurocrit Care* 2016;24:6-46.

16. Intercollegiate stroke working party. National clinical guideline for stroke. London, UK: Royal College of Physicians 2016.
17. Desborough MJ, Oakland KA, Landoni G, et al. Desmopressin for treatment of platelet dysfunction and reversal of antiplatelet agents: a systematic review and meta-analysis of randomized controlled trials. *J Thromb Haemost* 2017;15:263-72.
18. Desborough MJ, Oakland K, Brierley C, et al. Desmopressin for minimising perioperative blood transfusion. *Cochrane Database Syst Rev* 2017;7:CD001884
19. Naidech AM, Maas MB, Levasseur-Franklin KE, et al. Desmopressin improves platelet activity in acute intracerebral hemorrhage. *Stroke* 2014;45:2451-3.
20. Kapapa T, Rohrer S, Struve S, et al. Desmopressin acetate in intracranial haemorrhage. *Neurol Res Int* 2014;2014:298767.
21. Feldman EA, Meola G, Zyck S, et al. Retrospective assessment of desmopressin effectiveness and safety in patients with antiplatelet-associated intracerebral hemorrhage. *Crit Care Med* 2019;47:1759-65.
22. Schmidt KJ, Sager B, Zachariah J, et al. Cohort analysis of desmopressin effect on haematoma expansion in patients with spontaneous intracerebral hemorrhage and documented pre-ictus antiplatelet use. *J Clin Neurosci* 2019;66:33-7.
23. Weir CJ, Lees KR. Comparison of stratification and adaptive methods for treatment allocation in an acute stroke clinical trial. *Stat Med* 2003;22:705-26.
24. Bath PM, May J, Flaherty K, et al. Remote Assessment of Platelet Function in Patients with Acute Stroke or Transient Ischaemic Attack. *Stroke Res Treat* 2017;2017:7365684.
25. Moher D, Hopewell S, Schulz KF, et al. CONSORT 2010 explanation and elaboration: updated guidelines for reporting parallel group randomised trials. *BMJ* 2010;340:c869.
26. Schulz KF, Altman DG, Moher D. CONSORT 2010 statement: updated guidelines for reporting parallel group randomised trials. *BMJ* 2010;340:c332.
27. Sprigg N, Flaherty K, Appleton JP, et al. Tranexamic acid for hyperacute primary IntraCerebral Haemorrhage (TICH): an international randomised, placebo-controlled, phase 3 superiority trial. *Lancet* 2018;391:2107-15.

For peer review only

For peer review only - http://bmjopen.bmj.com/site/about/guidelines.xhtml

28. Baharoglu MI, Cordonnier C, Al-Shahi Salman R, et al. Platelet transfusion versus standard care after acute stroke due to spontaneous cerebral haemorrhage associated with antiplatelet therapy (PATCH): a randomised, open- label, phase 3 trial. *Lancet* 2016;387:2605-13.

For peer review only

For peer review only - http://bmjopen.bmj.com/site/about/guidelines.xhtml

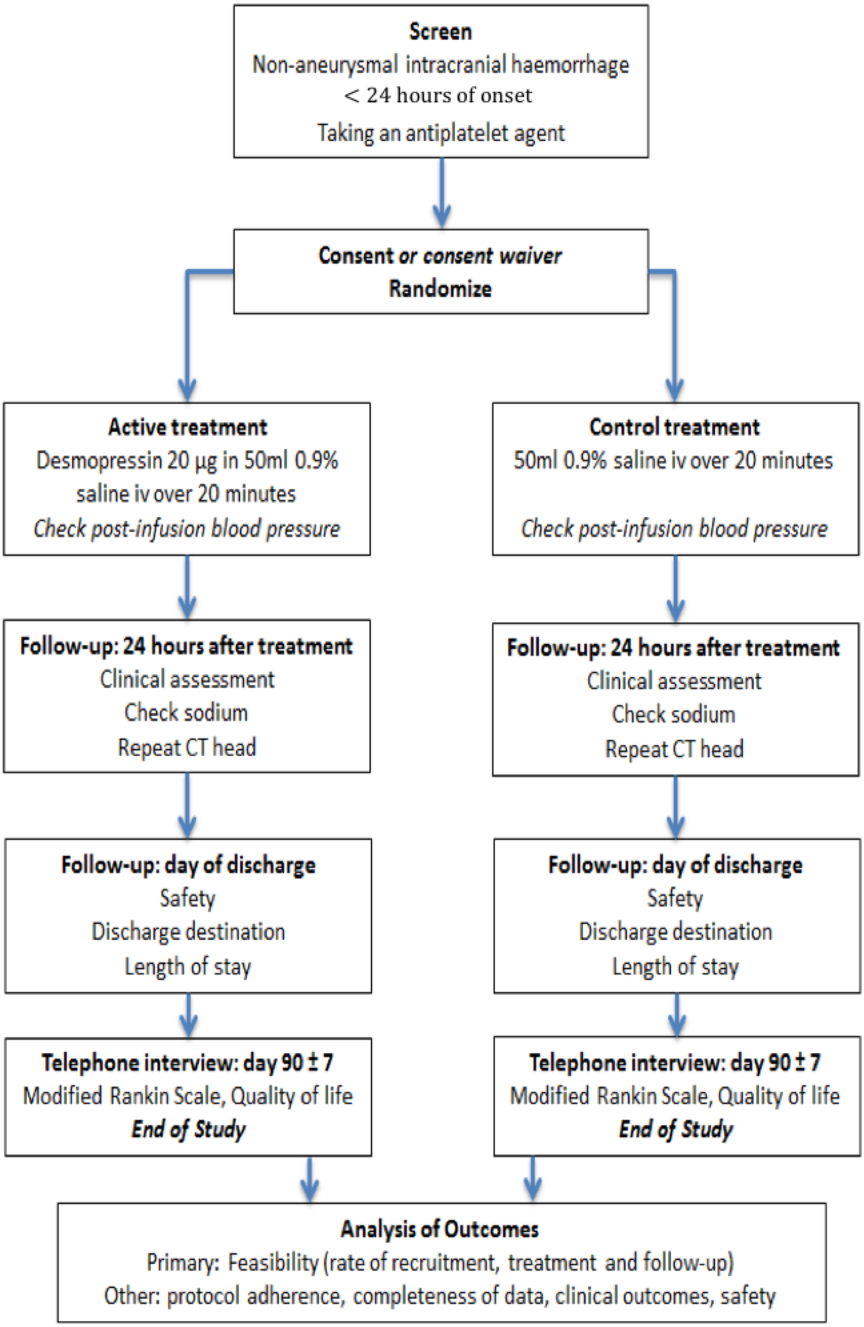

For peer review only - http://bmjopen.bmj.com/site/about/guidelines.xhtml

(Form to be printed on local headed paper)

PARTICIPANT CONSENT FORM

(Final version 2.0: 26 August 2019)

Title of Study: Desmopressin for reversal of Antiplatelet drugs in Stroke due to Haemorrhage (DASH)

IRAS Project ID: 233744. **CTA ref : 03057/0070/001-0001**

Name of Researcher:

Name of Participant:

Please initial box

1. I confirm that I have read and understand the information sheet version number 2.0: 26 August 2019 for the above study and have had the opportunity to ask questions. ☐
2. I understand that my participation is voluntary and that I am free to withdraw at any time, without giving any reason, and without my medical care or legal rights being affected. I understand that should I withdraw, then the information collected so far cannot be erased and that this information may still be used in the project analysis. ☐
3. I understand that relevant sections of my medical notes and data collected in the study may be looked at by authorised individuals from the University of Nottingham, the research group and regulatory authorities where it is relevant to my taking part in this study. I give permission for these individuals to have access to these records and to collect, store, analyse and publish information obtained from my participation in this study. I understand that my personal details will be kept confidential. ☐
4. I understand and agree that blood samples will be taken for analysis to monitor the effects of treatment given in DASH. Samples will be destroyed after analysis. ☐
5. I understand that the information held and maintained by the NHS Digital and other central UK NHS bodies may be used to help contact me or provide information about my health status. ☐
6. I agree to my GP being informed of my participation in this study and providing information about my health status and contact details if needed. ☐
7. I agree to take part in the above study. ☐

________________________	______________	____________________
Name of Participant	Date	Signature
________________________	______________	____________________
Name of Person taking consent	Date	Signature

PARTICIPANT CONSENT FORM - (Final version 2.0: 26 August 2019**)**

3 copies: 1 for participant, 1 for the project notes and 1 for the medical notes

For peer review only

Local Letterhead to be added

Participant Information Sheet
(Final version 2.0: 26 August 2019)

IRAS Project ID: 233744

Title of Study: Desmopressin for reversal of Antiplatelet drugs in Stroke due to Haemorrhage (DASH)

Name of Chief Investigator: Prof Nikola Sprigg
Local Researcher(s): xxxxxxxxxxxxxxxxxxxxxxxxx

We would like to invite you to take part in our research study. Before you decide we would like you to understand why the research is being done and what it would involve for you. One of our team will go through the information sheet with you and answer any questions you have. Talk to others about the study if you wish. Ask us if there is anything that is not clear, or if you would like more information.

What is the purpose of the study?

When someone has a stroke caused by bleeding into the brain (haemorrhagic stroke) permanent brain damage can occur and result in long term disability. There is also a chance that the bleeding can increase, which may cause worse disability or be life threatening. This happens in approximately 20-30% of haemorrhagic stroke patients. Bleeding is worse for patients taking drugs such as aspirin or clopidogrel (anti-platelet drugs).

At present, there is no available treatment that is effective at reducing the bleeding in the brain and improving the recovery. New treatments are being developed to treat stroke, but it can be very hard to test whether they work in the first few hours because often patients take longer than this to get to hospital and have investigations such as brain scanning. Also, some treatments are not suitable for all patients. This can make testing new treatments difficult.

In this trial, we want to test whether it is possible to give a drug (desmopressin) to patients within 24 hours after a haemorrhagic stroke. Continued or increased bleeding into the brain (so called haematoma expansion) is not uncommon in the first hours and days following a haemorrhagic stroke and increases the risk of the patient not recovering fully and being left with some disability. Stopping the bleeding in the first hours and days after stroke with medications might help patients to recover better. The treatment we are testing is a drug that encourages blood to clot - to stop bleeding. We hope that we will be able to show that giving the drug may reduce the chances of dying and being left with disability after a haemorrhagic stroke. This drug is not given routinely after stroke.

We aim to assess in this trial what effect desmopressin has on bleeding after a haemorrhagic stroke. Desmopressin is a tried and tested drug in other medical conditions that acts quickly to help the blood to clot and stop bleeding.

In order to do a proper comparison, we need to give some people the active drug and some people a dummy (placebo) treatment. In this trial, the dummy treatment is salt water. Half of the patients in the trial will receive the drug desmopressin and half will have placebo treatment.

For peer review only - http://bmjopen.bmj.com/site/about/guidelines.xhtml

The data will help doctors decide whether blood thickening treatments like desmopressin can be used in patients with acute haemorrhagic strokes who are taking anti-platelet drugs to try to prevent death and improve recovery.

Why have I been invited?

You are being invited to take part because you have had a stroke caused by bleeding into the brain – this is called a haemorrhagic stroke. We are inviting 50 participants like you to take part in the study.

Do I have to take part?

It is up to you to decide whether or not to take part. If you do decide to take part you will be given this information sheet to keep and be asked to sign a consent form. If you decide to take part you are still free to withdraw at any time and without giving a reason. This would not affect your legal rights.

What will happen to me if I take part?

Your involvement in the study will last for 3 months. In this study, the treatment (either desmopressin or dummy) is given as a drip via a cannula (needle inserted into a vein, usually into the back of the hand), which will be given over approximately 20 minutes.

We select which treatment you receive randomly (like tossing a coin) because this is how most clinical trials are carried out. When we don't know whether a treatment is effective or not we need to test it against not getting that treatment (called a control). Using randomisation to put patients into treatment groups is the best way to get a true answer as to whether a treatment works or not. In this trial, it is important to test whether the drug desmopressin can be given at random in a trial.

The treatment (either desmopressin or dummy) will be given a drip as soon as possible once you have decided you wish to take part in the study. The treatment will be given via a drip over approximately 20 minutes. You will not know if you received the drug or the dummy. The treatment will be given once, and then the treatment will stop.

We will take 3 blood samples from the vein in your arm. One before we start the study treatment to look at your blood clotting status, one immediately after and another 24 hours after treatment to check the salt levels in your blood stream. Wherever possible these will be taken with any routine blood samples your doctor asks for.

During the next 7 days, a nurse will check your condition looking in particular for signs of side effects of the treatment. We will also repeat a brain scan the day after the treatment to assess effects of the treatment. The brain scan will last less than 5 minutes but you will need to go to the x-ray department for the brain scan, which may take approximately an hour.

We ask your permission to contact your GP or check with the NHS Information Centre to check on your condition three months after your stroke and to confirm your contact details. You will then be contacted for a telephone consultation with a member of the research team, this can also be conducted by postal questionnaire if you prefer. This is to check your condition at that time. It will involve asking how you are able to move around, about how you feel your life has been affected by the stroke and some brief memory tests. In order to make the final evaluation of the study as objective as possible, the person who telephones you will not know if you received the active treatment or not.

Other than described here, your treatment will be exactly the same as for all stroke patients.

For peer review only - http://bmjopen.bmj.com/site/about/guidelines.xhtml

Expenses and payments

Participants will not be paid to participate in the study. There will be no charge for the trial medication. Travel expenses will be offered for any visits incurred as a result of participation.

What are the possible disadvantages and risks of taking part?

Treatment with any drugs can result in possible side effects and the side effects from desmopressin are generally mild. They can include headache, abdominal pain, low blood pressure and dizziness. The drug can increase the risk of seizures but this is very rare.

However, because the treatment works by stopping bleeding there is a chance it can cause an increase in blood clot formation. This can occur in the legs (deep vein thrombosis, DVT) or the lungs (Pulmonary embolism, PE) and is potentially very serious and maybe even life threatening. If you have previously suffered from blood clots in the legs or lungs you may not be able to participate in this study.

In 65 previous studies where desmopressin has been used to reduce bleeding during operations, desmopressin was safe. There was no increase in serious side effects, such as blood clots, in the patients who were treated with desmopressin.

Because desmopressin is already routinely used in a number of bleeding conditions, we expect the potential benefit of the drug (stopping bleeding in to the brain) to outweigh the low risk of serious side effects (such as blood clots). However, we do not know this for certain and will monitor all participants closely for side effects.

You must inform your doctor or member of the research team if you feel you have had a reaction to the medication.

We will take 3 blood samples from the vein in your arm, this can cause mild discomfort/pain and slight bruising.

You will have an extra CT brain scan performed as part of this trial. This is exactly the same as the CT scan that you had when you first came to hospital. The scan itself takes less than 5 minutes and does not involve any injections. The scan uses x-rays, which in large amounts can be harmful, but for this extra CT head scan the additional risk to you from the scan has been judged to be extremely small and is comparable with the annual risk of dying from an accident in the home.

You will need to be followed up by the research team for 3 months after starting the study.

What are the possible benefits of taking part?

Your participation in this study may reduce the symptoms of your haemorrhagic stroke or improve long-term recovery. However, we cannot promise the study will help you, and participation is voluntary. The information we get from your involvement may benefit other people who may have a stroke in the future.

What happens when the research study stops?

We aim to treat 50 patients in this study from the UK. When it has finished, we will look at the data and decide whether the treatment could be used for more patients with haemorrhagic stroke. We may not directly tell you or your relative the results of the study, but they will be published in a journal where they can be read.

For peer review only - http://bmjopen.bmj.com/site/about/guidelines.xhtml

Will my taking part in the study be kept confidential?

We will follow ethical and legal practice and all information about you will be handled in confidence.

If you join the study, we will use information collected from you [and your medical records] during the course of the research. This information will be kept **strictly confidential**, stored in a secure and locked office, and on a password protected database at the University of Nottingham. Under UK Data Protection laws the University is the Data Controller (legally responsible for the data security) and the Chief Investigator of this study (named above) is the Data Custodian (manages access to the data). This means we are responsible for looking after your information and using it properly. Your rights to access, change or move your information are limited as we need to manage your information in specific ways to comply with certain laws and for the research to be reliable and accurate. To safeguard your rights we will use the minimum personally – identifiable information possible.

You can find out more about how we use your information and to read our privacy notice at: https://www.nottingham.ac.uk/utilities/privacy.aspx. A hard copy can be made available on request.

The data collected for the study will be looked at and stored by authorised persons from the University of Nottingham who are organising the research. They may also be looked at by authorised people from regulatory organisations to check that the study is being carried out correctly. All will have a duty of confidentiality to you as a research participant and we will do our best to meet this duty.

Where possible information about you which leaves the site will have your name and address removed and a unique code will be used so that you cannot be recognised from it. However sometimes we need to ensure that we can recognise you to link the research data with your medical records so in these instances we will need to know your name and date of birth. We will also need this information if we need to follow up your medical records as part of the research, where we may need to ask the Government services that hold medical information about you (such as NHS Digital, the Office for National Statistics, among others) to provide this information to us. By signing the consent form you agree to the above.

Your contact information will be kept by the University of Nottingham for 6 months after the end of the study so that we are able to contact you about the findings of the study and possible follow-up studies (unless you advise us that you do not wish to be contacted). This information will be kept separately from the research data collected and only those who need to will have access to it. All other data (research data) will be kept securely for 7 years. After this time your data will be disposed of securely. During this time all precautions will be taken by all those involved to maintain your confidentiality, only members of the research team given permission by the data custodian will have access to your personal data.

In accordance with the University of Nottingham's, the Government's and our funders' policies we may share our research data with researchers in other Universities and organisations, including those in other countries, for research in health and social care. Sharing research data is important to allow peer scrutiny, re-use (and therefore avoiding duplication of research) and to understand the bigger picture in particular areas of research. Data sharing in this way is usually anonymised (so that you could not be identified) but if we need to share identifiable information we will seek your consent for this and ensure it is secure. You will be made aware then if the data is to be shared with

For peer review only - http://bmjopen.bmj.com/site/about/guidelines.xhtml

countries whose data protection laws differ to those of the UK and how we will protect your confidentiality.

Although what you say to us is confidential, should you disclose anything to us which we feel puts you or anyone else at any risk, we may feel it necessary to report this to the appropriate persons.

What will happen if I don't want to carry on with the study?

Your participation is voluntary and you are free to withdraw at any time, without giving any reason, and without your legal rights being affected. If you withdraw we will no longer collect any information about you or from you. We will keep the information about you that we have already obtained as we are not allowed to tamper with study records and this information may have already been used in some analyses and may still be used in the final study analyses.

Involvement of the General Practitioner/Family doctor (GP)

If you are enrolled in the study we will inform your General Practitioner.

What will happen to any samples I give?

The samples will be stored with a code unique to you and securely at the University of Nottingham and at The Oxford Haemophilia and Thrombosis Centre under the University's Human Tissue Research Licence (no 12265). At the end of the study samples will be destroyed in accordance with the Human Tissue Act guidelines.

Will any genetic tests be done?

No, we will not be collecting any genetic samples.

What will happen to the results of the research study?

The results of the research may be published. If so, this will be in a medical journal. You will not be identified in any report.

Who is organising and funding the research?

This research is being organised by the University of Nottingham and is being funded by NIHR RfPB.

Who has reviewed the study?

All research in healthcare is looked at by independent group of people, called a Research Ethics Committee, to protect your interests. This study has been reviewed and given favourable opinion by the local Ethics Committee [*NHS HRA East Midlands – Nottingham 2*]

Who should be contacted if there is a problem?

If you have a concern about any aspect of this study, you should ask to speak to the researchers who will do their best to answer your questions. The researchers' contact details are given at the end of this information sheet. If you remain unhappy and wish to complain formally, you can do this by contacting the [please provide below the contact details of PALS for the local hospital]

For peer review only - http://bmjopen.bmj.com/site/about/guidelines.xhtml

In the event that something does go wrong and you are harmed during the research and this is due to someone's negligence then you may have grounds for a legal action for compensation against the University of Nottingham but you may have to pay your legal costs. The normal National Health Service complaints mechanisms will still be available to you.

Further information and contact details

Trial / Study Coordinating Centre:
Division of Clinical Neuroscience, Stroke
University of Nottingham
Hucknall Road
Nottingham
NG5 1PB
Phone: 0115 8231770
Chief Investigator:
Prof Nikola Sprigg
Phone:0115 8231765

Contact details of PALS for the local hospital:

For peer review only

For peer review only - http://bmjopen.bmj.com/site/about/guidelines.xhtml

University of Nottingham
UK | CHINA | MALAYSIA

DASH: Desmopressin for reversal of Antiplatelet drugs in Stroke due to Haemorrhage

IRAS Project ID: 233744 **CTA ref: 03057/0070/001-0001**

Name of Researcher: ______________________________

Name of Participant: ______________________________

I confirm that I have been given a copy of the Patient Information Sheet (Version 2.0 dated 11 Jan 2019) and I agree that I /my relative or friend / this stroke patient (delete as appropriate)

- Will take part in the DASH study
- For my medical records to be accessed, blood samples to be taken and stored and brain scans used by the study team
- To be followed up at 3 months
- For my GP to be informed
- For my contact details to be collected and used for the purpose of the study
- For my confidential data to be used in further research analysis about ICH.

I understand that I am free to withdraw from the study at any point without giving a reason.

Patient consent – to be completed if participant has capacity to consent

Name of Participant	Date	Signature
Name of Person taking consent	Date	Signature
Name of Witness if participant unable to physically sign	Date	Signature

Personal or professional nominee consent - to be completed if participant does not have capacity to consent

Name of Person giving nominee consent	Date	Signature
Relationship to patient (please tick):	Relative/carer/friend ☐	Healthcare Professional ☐
Name of Person taking consent	Date	Signature
**Telemedicine used** (please tick if Yes) ☐		
Name of Witness if consent taken [illegible]	Date	Signature

For peer review only - http://bmjopen.bmj.com/site/about/guidelines.xhtml

DASH Research Study – Information Sheet

Final version 2.0 – 11 Jan 2019

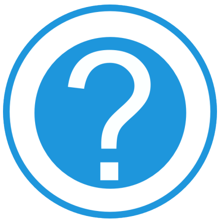

What is this about?

- We want to know if you would like you to take part in a research study called DASH. DASH will test the effect of the drug called desmopressin that may stop bleeding by reversing the effect of blood thinning medication.
- Research staff will discuss the study with you and can answer any questions you may have. If you are not well enough we will try to ask your family or friend. If they are not present an independent doctor will decide for you on your behalf.
- Taking part in the study is voluntary; you don't have to take part.

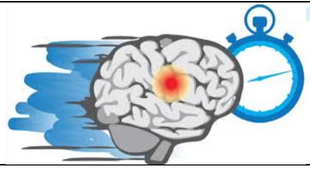

Why are we asking you to take part in the study?

- You have had a stroke caused by bleeding in the brain and you have been taking blood thinning medication.

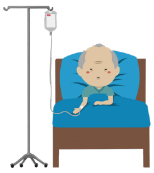

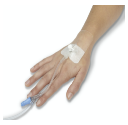

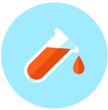

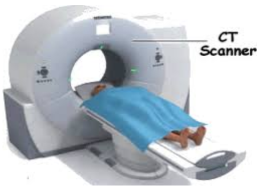

If you take part:

- You will receive all the care and treatments you would normally receive.
- The medical team will give you a drip into your vein via a small needle, which takes 20 minutes.
- Half the people in this study will get a drip containing desmopressin and half will get a drip containing no drug (placebo).
- Which drip you are given is decided by chance (like flipping a coin). You won't know which drip you will have been given.
- You will have three blood tests: one before the drip, one immediately after and another one on the next day to monitor results of the drip.
- You will have an extra head scan at some point in the next 2 days to monitor the bleeding. The study team will have copies of your brain scans.

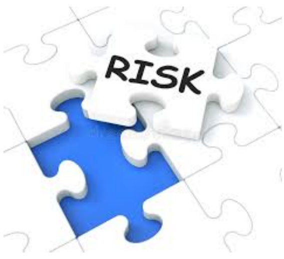

Risks

The drug, desmopressin, has been given safely to thousands of patients with inherited bleeding problems.

- The drug can cause mild side effects: headache, nausea and vomiting, which can all be easily treated. Very rarely it can cause more serious sides effects and we will monitor closely for this.
- The extra head scan has around the same amount of radiation as living for a year in the UK.

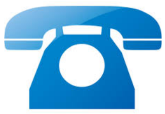

90 days after your stroke:

- A researcher will call you to see how you are, if you have had any problems and how well you have recovered.
- If you are not well enough to talk we will try to ask your family, friend or GP.

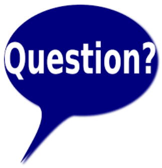

During the study:

- If you have any questions then please ask.
- You may decide you do not want to take part at any time. This will not affect your care now or in the future.
- All the information we hold about you (including brain scans) will be kept in the strictest confidence.

Short patient information sheet – pictorial - Final v2.0 – 11 Jan 2019

For peer review only

STANDARD PROTOCOL ITEMS: RECOMMENDATIONS FOR INTERVENTIONAL TRIALS

SPIRIT 2013 Checklist: Recommended items to address in a clinical trial protocol and related documents*

Section/item	Item No	Description
**Administrative information**		
Title	1	Descriptive title identifying the study design, population, interventions, and, if applicable, trial acronym **Page 1**
Trial registration	2a	Trial identifier and registry name. If not yet registered, name of intended registry **Page 3**
	2b	All items from the World Health Organization Trial Registration Data Set **Page 3**
Protocol version	3	Date and version identifier **Page 3**
Funding	4	Sources and types of financial, material, and other support **Page 4**
Roles and responsibilities	5a	Names, affiliations, and roles of protocol contributors **Page 1 and page 17**
	5b	Name and contact information for the trial sponsor **Page 15**
	5c	Role of study sponsor and funders, if any, in study design; collection, management, analysis, and interpretation of data; writing of the report; and the decision to submit the report for publication, including whether they will have ultimate authority over any of these activities **Page 15**
	5d	Composition, roles, and responsibilities of the coordinating centre, steering committee, endpoint adjudication committee, data management team, and other individuals or groups overseeing the trial, if applicable (see Item 21a for data monitoring committee) **Pages 15-17**

For peer review only

Introduction

Background and rationale	6a	Description of research question and justification for undertaking the trial, including summary of relevant studies (published and unpublished) examining benefits and harms for each intervention **Page 2**
	6b	Explanation for choice of comparators **Page 8**
Objectives	7	Specific objectives or hypotheses **Page 6**
Trial design	8	Description of trial design including type of trial (eg, parallel group, crossover, factorial, single group), allocation ratio, and framework (eg, superiority, equivalence, noninferiority, exploratory) **Page 6**

Methods: Participants, interventions, and outcomes

Study setting	9	Description of study settings (eg, community clinic, academic hospital) and list of countries where data will be collected. Reference to where list of study sites can be obtained **Page 17**
Eligibility criteria	10	Inclusion and exclusion criteria for participants. If applicable, eligibility criteria for study centres and individuals who will perform the interventions (eg, surgeons, psychotherapists) **Page 6**
Interventions	11a	Interventions for each group with sufficient detail to allow replication, including how and when they will be administered **Page 8**
	11b	Criteria for discontinuing or modifying allocated interventions for a given trial participant (eg, drug dose change in response to harms, participant request, or improving/worsening disease) **N/A – single treatment only**
	11c	Strategies to improve adherence to intervention protocols, and any procedures for monitoring adherence (eg, drug tablet return, laboratory tests) **N/A – single treatment only**
	11d	Relevant concomitant care and interventions that are permitted or prohibited during the trial **Page 8**

For peer

Outcomes	12	Primary, secondary, and other outcomes, including the specific measurement variable (eg, systolic blood pressure), analysis metric (eg, change from baseline, final value, time to event), method of aggregation (eg, median, proportion), and time point for each outcome. Explanation of the clinical relevance of chosen efficacy and harm outcomes is strongly recommended<br>**Page 9-10**
Participant timeline	13	Time schedule of enrolment, interventions (including any run-ins and washouts), assessments, and visits for participants. A schematic diagram is highly recommended (see Figure)<br>**Figure 1**
Sample size	14	Estimated number of participants needed to achieve study objectives and how it was determined, including clinical and statistical assumptions supporting any sample size calculations<br>**Page 12**
Recruitment	15	Strategies for achieving adequate participant enrolment to reach target sample size<br>**This is a feasibility trial**

Methods: Assignment of interventions (for controlled trials)

Allocation:

Sequence generation	16a	Method of generating the allocation sequence (eg, computer-generated random numbers), and list of any factors for stratification. To reduce predictability of a random sequence, details of any planned restriction (eg, blocking) should be provided in a separate document that is unavailable to those who enrol participants or assign interventions<br>**Page 7**
Allocation concealment mechanism	16b	Mechanism of implementing the allocation sequence (eg, central telephone; sequentially numbered, opaque, sealed envelopes), describing any steps to conceal the sequence until interventions are assigned<br>**Page 7**
Implementation	16c	Who will generate the allocation sequence, who will enrol participants, and who will assign participants to interventions<br>**Page 7**
Blinding (masking)	17a	Who will be blinded after assignment to interventions (eg, trial participants, care providers, outcome assessors, data analysts), and how<br>**Page 7**

	17b	If blinded, circumstances under which unblinding is permissible, and procedure for revealing a participant's allocated intervention during the trial<br>**Page 7**

Methods: Data collection, management, and analysis

Data collection methods	18a	Plans for assessment and collection of outcome, baseline, and other trial data, including any related processes to promote data quality (eg, duplicate measurements, training of assessors) and a description of study instruments (eg, questionnaires, laboratory tests) along with their reliability and validity, if known. Reference to where data collection forms can be found, if not in the protocol<br>**Page 8**
	18b	Plans to promote participant retention and complete follow-up, including list of any outcome data to be collected for participants who discontinue or deviate from intervention protocols<br>**N/A – single treatment only**
Data management	19	Plans for data entry, coding, security, and storage, including any related processes to promote data quality (eg, double data entry; range checks for data values). Reference to where details of data management procedures can be found, if not in the protocol<br>**Page 12**
Statistical methods	20a	Statistical methods for analysing primary and secondary outcomes. Reference to where other details of the statistical analysis plan can be found, if not in the protocol<br>**Page 10**
	20b	Methods for any additional analyses (eg, subgroup and adjusted analyses)<br>N/A
	20c	Definition of analysis population relating to protocol non-adherence (eg, as randomised analysis), and any statistical methods to handle missing data (eg, multiple imputation)<br>**Page 11**

Methods: Monitoring

Data monitoring	21a	Composition of data monitoring committee (DMC); summary of its role and reporting structure; statement of whether it is independent from the sponsor and competing interests; and reference to where further details about its charter can be found, if not in the protocol. Alternatively, an explanation of why a DMC is not needed<br>**Page 16**

	21b	Description of any interim analyses and stopping guidelines, including who will have access to these interim results and make the final decision to terminate the trial<br>**Page 17**
Harms	22	Plans for collecting, assessing, reporting, and managing solicited and spontaneously reported adverse events and other unintended effects of trial interventions or trial conduct<br>**Page 10**
Auditing	23	Frequency and procedures for auditing trial conduct, if any, and whether the process will be independent from investigators and the sponsor<br>**Page 16**
**Ethics and dissemination**		
Research ethics approval	24	Plans for seeking research ethics committee/institutional review board (REC/IRB) approval<br>**Page 12**
Protocol amendments	25	Plans for communicating important protocol modifications (eg, changes to eligibility criteria, outcomes, analyses) to relevant parties (eg, investigators, REC/IRBs, trial participants, trial registries, journals, regulators)<br>**Page 13**
Consent or assent	26a	Who will obtain informed consent or assent from potential trial participants or authorised surrogates, and how (see Item 32)<br>**Page 7**
	26b	Additional consent provisions for collection and use of participant data and biological specimens in ancillary studies, if applicable<br>**N/A**
Confidentiality	27	How personal information about potential and enrolled participants will be collected, shared, and maintained in order to protect confidentiality before, during, and after the trial<br>**Page 13**
Declaration of interests	28	Financial and other competing interests for principal investigators for the overall trial and each study site<br>**Page 17**
Access to data	29	Statement of who will have access to the final trial dataset, and disclosure of contractual agreements that limit such access for investigators<br>**Page 13**

For peer review only

Ancillary and post-trial care	30	Provisions, if any, for ancillary and post-trial care, and for compensation to those who suffer harm from trial participation **Page 14**
Dissemination policy	31a	Plans for investigators and sponsor to communicate trial results to participants, healthcare professionals, the public, and other relevant groups (eg, via publication, reporting in results databases, or other data sharing arrangements), including any publication restrictions **Page 13**
	31b	Authorship eligibility guidelines and any intended use of professional writers **Not used**
	31c	Plans, if any, for granting public access to the full protocol, participant-level dataset, and statistical code **Not planned**
**Appendices**		
Informed consent materials	32	Model consent form and other related documentation given to participants and authorised surrogates **Supplementary material**
Biological specimens	33	Plans for collection, laboratory evaluation, and storage of biological specimens for genetic or molecular analysis in the current trial and for future use in ancillary studies, if applicable **N/A**

*It is strongly recommended that this checklist be read in conjunction with the SPIRIT 2013 Explanation & Elaboration for important clarification on the items. Amendments to the protocol should be tracked and dated. The SPIRIT checklist is copyrighted by the SPIRIT Group under the Creative Commons "Attribution-NonCommercial-NoDerivs 3.0 Unported" license.

For peer review only
